# Data-driven subphenotyping of severe ARDS patients requiring VV-ECMO

**DOI:** 10.3389/fdgth.2026.1736849

**Published:** 2026-07-03

**Authors:** Micha Landoll, Stephan Strassmann, Wolfram Windisch, Ulrich Steinseifer, Andreas Schuppert, Michael Neidlin, Christian Karagiannidis

**Affiliations:** 1 Department of Pneumology and Critical Care Medicine, ARDS and ECMO Centre, Cologne-Merheim Hospital, Cologne, Germany; 2 Cardiovascular Engineering, Applied Medical Engineering, RWTH Aachen University, Aachen, Germany; 3 Institute for Computational Biomedicine, RWTH Aachen University, Aachen, Germany; 4 Department of Respiratory Medicine, University Witten/Herdecke, Witten, Germany; 5 Center for Computational Life Sciences, RWTH Aachen University, Aachen, Germany

**Keywords:** ARDS, cluster analysis, electronic health records, subphenotypes, VV-ECMO

## Abstract

**Background:**

Acute respiratory distress syndrome is a heterogeneous syndrome that complicates risk stratification, therapy monitoring and personalised treatment during veno-venous extracorporeal membrane oxygenation. This study aims to identify discrete acute respiratory distress syndrome subphenotypes among veno-venous extracorporeal membrane oxygenation (VV-ECMO) patients using high-resolution electronic health record clustering, and to assess differences in clinical outcomes.

**Materials and methods:**

We conducted a study of 598 adult patients with acute respiratory distress syndrome treated with VV-ECMO. Twenty-six clinically relevant parameters spanning inflammation, coagulation, kidney/liver function, mechanical ventilation, and ECMO parameters were analysed using K-means clustering. Shapley Additive Explanations models were used to identify key differentiating parameters between clusters and independent survival factors. Clinical outcomes were compared between clusters, including treatment duration and survival rates in intensive care.

**Results:**

Cluster analysis revealed distinct subphenotypes primarily driven by differences in inflammation (procalcitonin and C-reactive protein), kidney/liver function (creatinine and urea), coagulation (fibrinogen and D-dimer), and mechanical ventilation (positive end-expiratory pressure). Survival rates varied considerably between clusters, most notably within the kidney/liver function (32%) and combined parameter categories (21%). Subphenotypes defined solely by ECMO or ventilator settings showed smaller differences. Intensive care unit length of stay was longer in clusters with multi-organ dysfunction.

**Conclusions:**

Early data-driven clustering of electronic health record parameters identifies clinically meaningful acute respiratory distress syndrome subphenotypes among veno-venous extracorporeal membrane oxygenation patients. Renal, hepatic, and inflammatory dysfunctions are critical determinants of survival. Subphenotype-based stratification may refine risk stratification and management in severe acute respiratory distress syndrome treated with VV-ECMO.

## Introduction

Acute respiratory distress syndrome (ARDS) is a life-threatening condition characterised by acute lung inflammation and increased pulmonary vascular permeability, leading to hypoxemia and acute respiratory failure (1–4). Veno-venous extracorporeal membrane oxygenation (VV-ECMO) has become a guideline-based therapy for the most severe cases (5–7). However, despite advances in ECMO technology, real-world mortality rates for ARDS patients requiring VV-ECMO remain high, exceeding 50% in some healthcare systems (7–10). Selecting patients and managing therapy are important clinical needs to improve ECMO outcomes (2, 11–16).

A major challenge in the management of ARDS is its heterogeneity. Patients present with different clinical features, underlying causes and responses to treatment (5, 11, 17, 18). Therefore, previous studies, such as those by Calfee et al. and Liu et al., have identified subphenotypes within the ARDS population, including hyper- and hypoinflammatory types, with different clinical outcomes and responses to treatment (19–22). For example, although hyperinflammatory patients generally have higher mortality, they tend to have more pronounced physiologic benefits from interventions such as higher positive end-expiratory pressure (PEEP), prone positioning or may respond better to pharmaceutical treatment (11, 23, 24). These findings highlight the importance of subphenotype-based ARDS management aimed at improving patient outcomes and pave the way for individualised treatment. Recent work by Nishikimi et al. has extended this approach to the most critically ill ARDS patients receiving VV-ECMO, identifying novel subphenotypes with different mortality rates and PEEP responsiveness (25). However, as the primary indications for ECMO initiation, such as low PaO_2_ and/or uncontrolled PaCO_2_, might be independent of the underlying ARDS origin, these general ARDS subphenotypes cannot be directly applied to guide post-cannulation management in ECMO patients. Furthermore, although studies using routinely available clinical data have reproducibly identified several ARDS subphenotypes, a dedicated phenotyping approach optimised for VV-ECMO patients is still lacking.

Deriving such subphenotypes from the vast amount of clinical data contained in electronic health records (EHRs) is challenging using conventional statistical methods due to the complexity of the data. Artificial intelligence and machine learning techniques, therefore, offer an attractive solution. For example, Stephens et al. have demonstrated the potential of deep learning to predict mortality in ECMO patients using data from the ELSO registry (26, 27). Similarly, Castela Forte et al. employed deep embedded clustering to identify and characterize high-risk clusters in a heterogeneous intensive care unit (ICU) population (28). Furthermore, Wang et al. developed a methodological pipeline to estimate unmeasured physiological parameters, producing high-fidelity, personalized phenotypes data from electronic health records (29).

This study aims to identify clinically meaningful post-cannulation subphenotypes of ARDS patients requiring VV-ECMO using routinely collected high-resolution electronic health record data and cluster analysis. The resulting subphenotypes will be further evaluated in terms of ICU survival and length of stay to refine risk stratification for therapy monitoring and adjustments.

## Materials and methods

### Study design and population

This single-centre analysis uses EHRs from the ARDS and ECMO Centre at Cologne-Merheim, University of Witten/Herdecke. The study cohort includes ECMO patients diagnosed with ARDS and patients with bacterial or viral pneumonia. All patients meet the physiological criteria for severe respiratory failure as defined by the Berlin definition (30). These patients were admitted to the ICU between October 2012 and September 2023, most following inter-hospital transfer. In this referral centre, patients either arrived with VV-ECMO already in place following brief cannulation by a mobile ECMO team, or were cannulated immediately upon admission. ECMO eligibility was determined primarily in accordance with the criteria of the EOLIA trial (7). The inclusion criteria encompassed patients with an ICD-coded diagnosis that was verified manually via a review of their clinical records. This was done to confirm that all patients fulfilled the Berlin criteria for ARDS, regardless of their primary diagnostic label, due to uncertainty surrounding billing labels in EHR. Patients who fulfilled these criteria but were ultimately managed without cannulation at the referring hospitals could not be traced in our records and were therefore not included. Exclusion criteria encompassed ICU stays under one day, substantial missing data in patient records, ECMO configurations other than VV-ECMO and patients under 18 years of age. The Medical Ethics Committee of Witten-Herdecke University approved the study (102/2019), conducted according to the Declaration of Helsinki and Good Clinical Practice principles.

### Data preparation from electronic health records

A systematic pipeline was used to extract and process data from the EHRs (Supplementary Figure E1). Instead of manually correcting individual patient records, we applied data cleaning functions systematically across the entire cohort dataset. The dataset included demographic information, clinical parameters, laboratory results, ventilator settings, ECMO parameters, treatment outcomes and physician annotations, excluding imaging data. In this cohort patients were eligible for ECMO at admission or arrived with external cannulation in place. The initial 48 h of ECMO initiation or, if not available, ICU admission were used to represent baseline characteristics, with outcomes analysed separately later to ensure quality and completeness. Thus, this study window reflects the early post-cannulation stabilisation phase. Median values were calculated to minimise transient variations and outliers. Parameters were retained if missingness was less than 23%. This threshold was chosen empirically based on a clear drop in data density in our cohort (see Supplementary Figure E2), and is consistent with common practices in EHR research (typically around 20%) (31, 32). Any remaining missing values were imputed using median values, in order to avoid introducing correlations between variables that would impose an artificial structure on the unsupervised clustering. However, norepinephrine was retained despite having 45.5% missing values, due to its high clinical relevance. Finally, extreme outliers were capped via winsorisation and min-max scaling was applied to ensure that all parameters contributed equally to Euclidean distances. This prevented high-variance markers from dominating and preserved relative magnitudes for clinical interpretation.

### Parameters selection

The final selection of key parameters for cluster analysis was guided by clinician expertise and literature, prioritising relevance, quality and availability. A Pearson correlation analysis (Supplementary Figure E3) was performed to identify and exclude redundant parameters, thereby ensuring the inclusion of the most informative parameters for clustering. Finally, twenty-six parameters across inflammation, coagulation, kidney and liver function, mechanical ventilation (MV) and ECMO parameters were included. Overall missingness across these parameters was negligible, with a median of 3.6% and an interquartile range of 2.5%–5.3% (Table 1).

**Table 1:** Summary of baseline characteristics of the study cohort during the first 48 h of VV-ECMO therapy. Continuous parameters are expressed as mean, 25th and 75th percentiles, and Boolean parameters are presented as percentages. Only parameters from the inflammation, kidney and liver function, coagulation, ECMO and mechanical ventilation categories were processed for clustering and imputed, while other parameters were descriptive only.

Category	Parameter	Missing values	Input para-meter	Survivor (*n* = 337)	Non Survivor (*n* = 261)	Entire Cohort (*n* = 598)
Demographics	Age (years)	0%		52.9 [45.9, 62.5]	56.4 [49.1, 65.5]	54.4 [46.5, 64.0]
	Sex (percent female)	0%		34%	31%	33%
	BMI	44.6%		31.2 [25.3, 34.6]	31.2 [24.7, 34.6]	31.2 [25.2, 34.6]
SOFA score	SOFA Score	62.4%		11.3 [10, 13]	13.6 [11, 16]	12.2 [10.8, 14]
	Component: Cardiovascular system	62.2%		2.8 [3, 3.1]	3.1 [3, 4]	2.9 [3, 4]
	Component: Coagulation	62.2%		0.35 [0, 0.50]	0.74 [0, 1.5]	0.50 [0, 1]
	Component: Liver	62.2%		0.42 [0, 1]	0.85 [0, 2]	0.59 [0, 1]
	Component: Renal function	62.2%		0.57 [0, 0.25]	1.5 [0, 3]	0.94 [0, 1.5]
	Component: Respiratory system	62.2%		3.7 [3.5, 4]	3.7 [3, 4]	3.7 [3, 4]
	Component: Central nervous system	62.2%		3.4 [4, 4]	3.7 [4, 4]	3.6 [4, 4]
Inflammation	Fluid balance hourly (mL/h)	0.3%	x	660.5 [209.8, 1,023.8]	804.0 [283.5, 1,398.2]	723.5 [251.5, 1,180.8]
	Leukocytes(10^3^/µL)	2.5%	x	13.0 [8.4, 16.8]	13.2 [8.5, 16.9]	13.1 [8.4, 16.8]
	Triglycerides (mg/dL)	2.5%	x	177.1 [111, 229]	179.6 [103, 231]	178.2 [108.5, 230.5]
	CRP (mg/L)	3.5%	x	17.3 [9.2, 25.1]	18.8 [12.4, 26.2]	18.0 [10.6, 25.6]
	PCT (ng/mL)	4.7%	x	5.2 [0.43, 7.7]	7.2 [1.1, 13.0]	6.1 [0.60, 9.8]
	Lactate, BGA (mmol/L)	2.7%	x	1.8 [1.2, 2.0]	2.5 [1.4, 2.9]	2.1 [1.3, 2.3]
	Albumin (g/dL)	3.8%	x	23.8 [21, 26]	22.7 [20, 25]	23.3 [20.5, 26]
	Norepinephrine (*μ*g/kg/min)	45.5%	x	0.12 [0.03, 0.17]	0.20 [0.06, 0.31]	0.15 [0.04, 0.20]
Inflammation and Kidney Liver	LDH (U/L)	4.3%	x	537.4 [345, 643]	594.2 [368.4, 704.8]	561.5 [352.1, 667.9]
Kidney Liver	GOT, AST (U/L)	5.5%	x	124.8 [39.4, 109.5]	174.0 [43.5, 160]	145.3 [41, 127.5]
	GPT, ALT (U/L)	4.8%	x	69.4 [23, 64]	85.8 [22.9, 79.9]	76.3 [23, 70]
	Urea (mg/dL)	2.5%	x	65.8 [35, 80.5]	91.1 [57, 115.5]	76.8 [40.5, 100.2]
	Creatinine, enzymatic (mg/dL)	3.7%	x	1.2 [0.64, 1.4]	1.8 [0.75, 2.5]	1.5 [0.67, 1.9]
	Bilirubin total (mg/dL)	2.0%	x	1.2 [0.51, 1.6]	1.9 [0.61, 2.4]	1.5 [0.56, 1.9]
Coagulation	INR	1.3%	x	1.1 [1.0, 1.2]	1.2 [1.1, 1.3]	1.2 [1.0, 1.2]
	PTT (s)	1.8%	x	45.0 [39, 49]	50.4 [43.5, 56]	47.4 [41, 52]
	D-dimers (ng/mL)	3.0%	x	9.3 [3, 12]	11.5 [4, 16]	10.3 [3, 14.5]
	Fibrinogen, Clauss (g/L)	2.8%	x	495.2 [367.9, 629.1]	490.6 [364, 636]	493.2 [366, 635.5]
ECMO	Pump speed (min^−1^)	2.2%	x	3,093.7 [2,600, 3,111.2]	3,033.6 [2,600, 3,060]	3,067.4 [2,600, 3,100]
	Blood flow (L/min)	1.7%	x	3.9 [3.5, 4.3]	3.8 [3.4, 4.3]	3.8 [3.5, 4.3]
	p. Art/ P3 (mmHg)	22.1%	x	140.1 [118, 162]	143.9 [123, 166]	141.8 [119.5, 164.4]
MV	Total Minute Ventilation (L/min)	13.2%	x	2.5 [1.5, 3.1]	2.7 [1.6, 3.1]	2.6 [1.6, 3.1]
	Ppeak (cmH_2_O)	4.2%	x	24.3 [21.8, 27.1]	25.5 [22.2, 28.6]	24.8 [22, 28]
	PEEP (cmH_2_O)	6.2%	x	13.7 [11, 16.0]	14.0 [11.1, 17]	13.8 [11, 16.9]
	Tidal Volume (mL)	13.2%	x	235.9 [170, 294.2]	219.0 [150, 264]	228.5 [160, 282]
	Respiratory Rate (min^−1^)	8%	x	10.9 [8, 13]	12.4 [10, 15]	11.5 [9, 14]
Outcome	ICU lenght of stay (days)	0%		35.0 [18, 44]	27.9 [9, 32]	31.9 [15, 42]
	ICU mortality (percent)	0%		0%	100%	44%
	ECMO duration (hours)	0%		555.2 [281, 695.8]	577.6 [163.5, 669]	565.1 [233.5, 692]
	MV duration (hours)	0.2%		431.1 [0, 677.5]	355.9 [0, 439.5]	397.9 [0, 616.8]

### Cluster analysis

The K-means clustering algorithm was selected despite its spherical cluster and equal-variance assumptions. These were mitigated through parameter-wise normalisation, which equalised feature ranges for balanced Euclidean distances. Its simple Euclidean framework ensures understandable centroids and computational scalability. Preliminary analyses show reasonable overlap with Gaussian mixture models and hierarchical clustering (Supplementary Figure E4), and exceptional internal stability (Supplementary Table E3) validates this. The Calinski-Harabasz index, silhouette analysis, and the elbow method were inspected per cluster category across the pre-specified range of two to five clusters (Supplementary Figure E5). We adopted three clusters across all categories as a deliberate study-design choice rather than a within-category metric optimum. A common cluster number was empirically feasible for each category and is consistent with the granularity of the established ARDS phenotype literature (19–21, 25).

### Interpretation models and assessment of clinical outcome

Centroid-based methods such as K-means often produce clusters that are difficult to interpret. This study used SHapley Additive exPlanations (SHAP) on a gradient boosting tree classifier (XGBoost) trained with the cluster labels to visualise parameters that distinguished between clusters and to identify the key factors influencing the cluster assignments. This approach transforms the interpretation of clustering results into a supervised problem and allows SHAP to be applied. In addition, an independent model was fitted to classify ICU survival, with SHAP applied to rank individual parameter contributions. This additional supervised model serves as a tool for interpreting which baseline features are most strongly associated with ICU survival. These SHAP attributions are model-dependent associations with cluster assignment and ICU survival, not causal or mechanistic drivers. Clinical outcomes, with ICU survival as the primary endpoint and ICU length of stay and ECMO duration as secondary endpoints, were then compared across the clusters in order to evaluate the clinical relevance of each subphenotype.

Between-cluster ICU outcome differences were assessed for each cluster category across three endpoints: ICU survival, ICU length of stay and ECMO duration. ICU survival was analysed using a Cox proportional hazards model, adjusted for age and sex. The cluster factor was evaluated using a likelihood-ratio test comparing the full model with the model adjusted only for age and sex, yielding one *p*-value per cluster category. ICU length of stay and ECMO duration were tested per cluster category using the Kruskal–Wallis test on the cluster groups. The resulting *p*-values were jointly corrected using the Benjamini–Hochberg false discovery rate procedure. Pairwise cluster hazard ratios for ICU mortality were derived from the Cox model with 95% Wald confidence intervals as descriptive effect estimates and were not separately corrected. Multivariate log-rank tests, cause-specific log-rank tests for ICU death and alive discharge, and scaled Schoenfeld residual checks (Bonferroni-aggregated across model terms) were additionally performed.

## Results

### Patient cohort and data preparation

**Figure 1:**
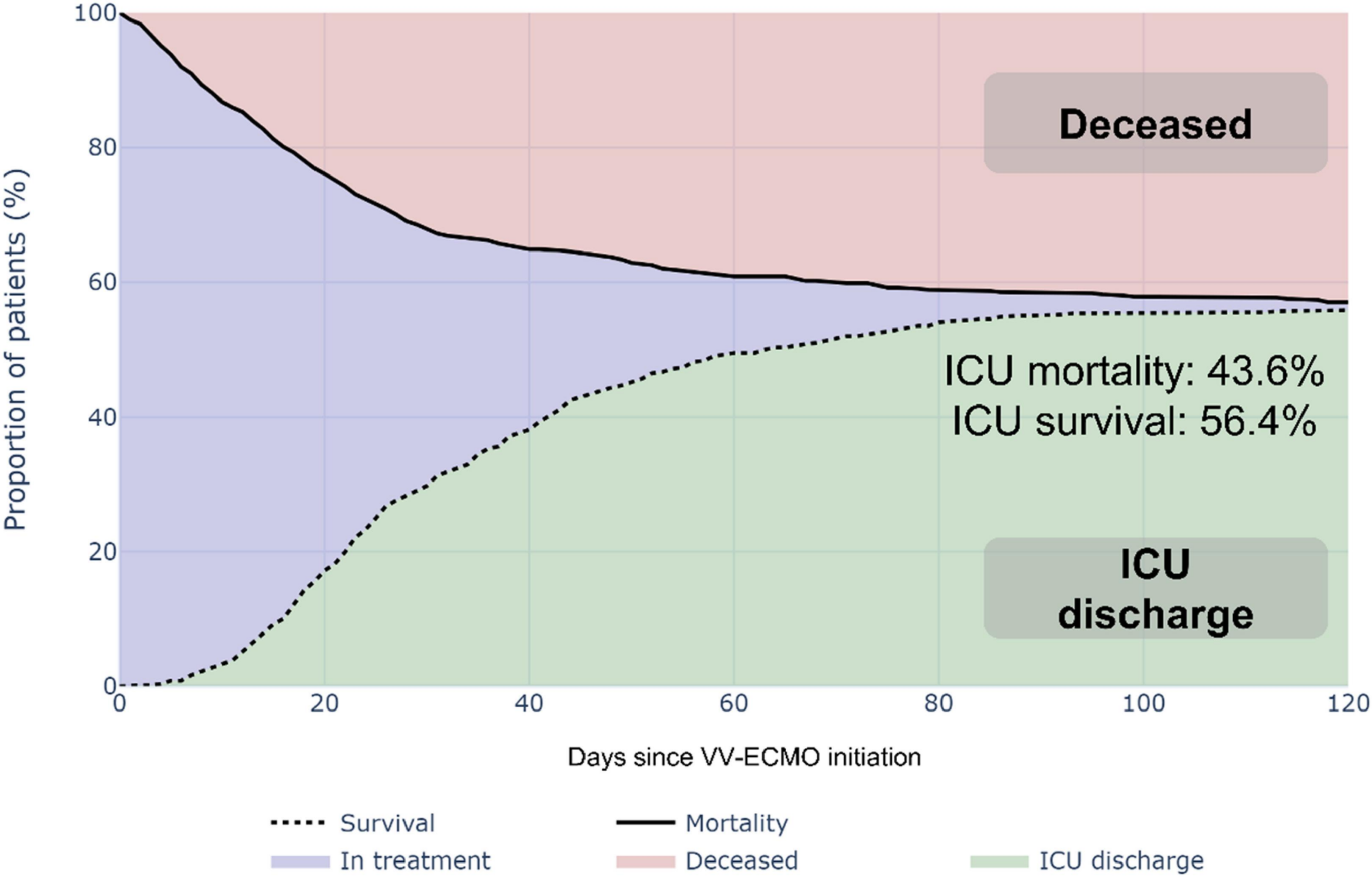
Cumulative incidence of ICU outcomes within 120 days after ECMO initiation. The purple area indicates patients still receiving ICU treatment, the green area represents patients discharged alive, and the red area represents patients who died in ICU. At any given time, the three areas together account for 100% of the patient cohort.

A total of 813 patients requiring ECMO support were screened, of whom 608 fulfilled ARDS diagnostic criteria per the Berlin definition. After application of exclusion criteria, the final cohort consisted of 598 patients with a complete data set admitted to the ICU of the ARDS and ECMO Centre Cologne-Merheim, as shown in Supplementary Figure E6. The mean age was 54.4 years (median: 56.9 years), with a male predominance of 67% and a body mass index of 31.2. The overall ICU survival rate was 56.4% with a mean ECMO duration of 23.5 days and an ICU length of stay of 31.9 days, as shown in Figure 1. The characteristics of the study cohort are presented in Table 1, Supplementary Figures E7 and E8. Additional characteristics comparing survivors and non-survivors are illustrated in Supplementary Figure E9.

### Cluster analysis of all parameters

**Figure 2:**
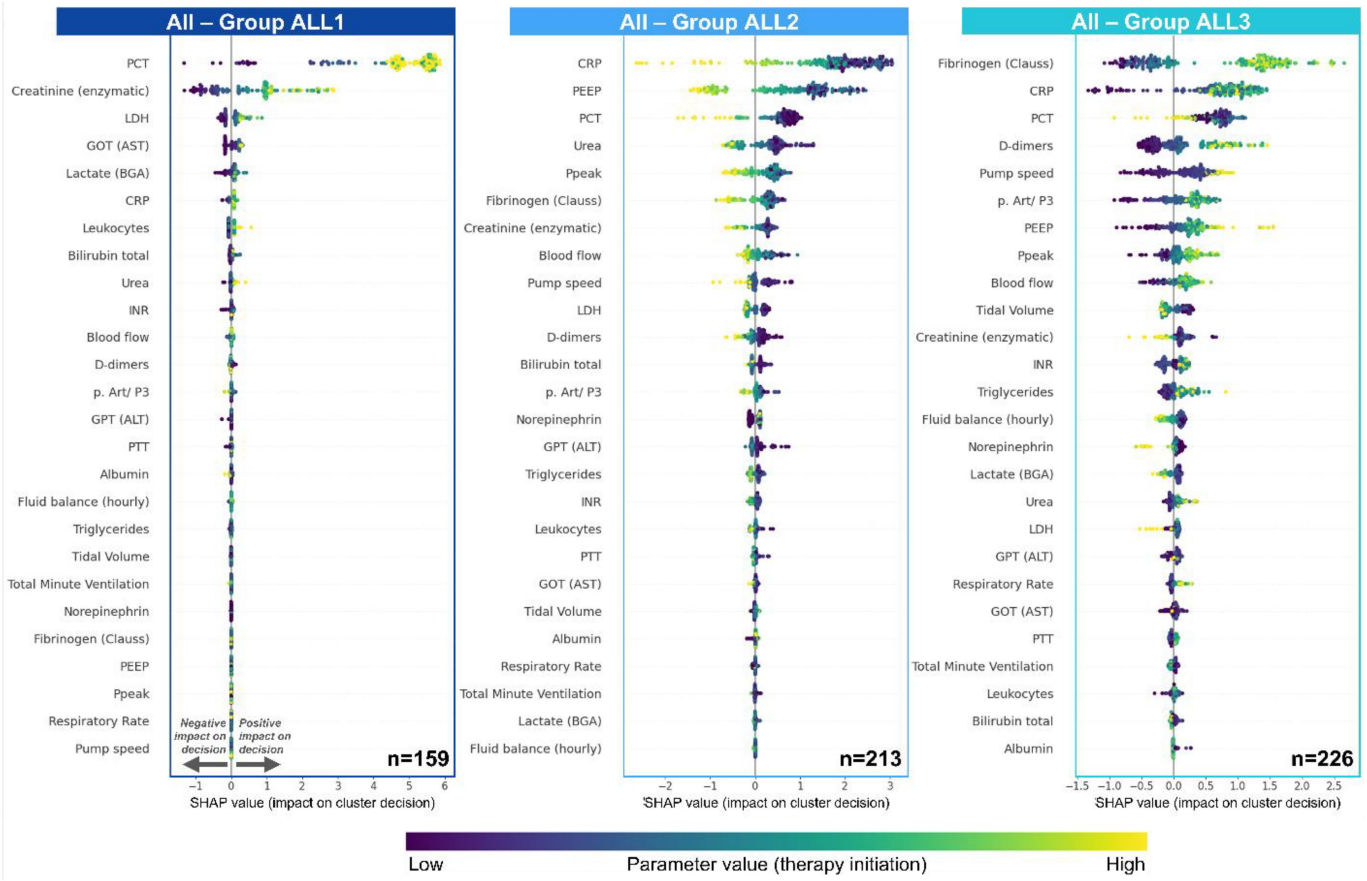
The SHAP summary bee swarm plot ranks clinical parameters by their impact on cluster assignments, with SHAP values on the *x*-axis indicating the magnitude and direction of each parameter's effect. Positive SHAP values increase the likelihood of assignment to a cluster, while negative values decrease it. Each dot represents a patient and is coloured according to the parameter value (from low in blue to high in yellow).

Figure 2 shows the identified clusters and the importance of each clinical parameter for clustering assignment in descending order. Additionally Supplementary Figure E10 summarises the importance of parameters for each clinical category. When considering all 26 parameters collectively, three clusters emerged with clinical profiles described in Supplementary Tables E1 and E2. The cluster group named *ALL1* was characterized by relatively elevated procalcitonin (PCT) and high creatinine levels. Cluster *ALL2* displayed lower values in C-reactive protein (CRP), PEEP, PCT, urea, and peak inspiratory pressure (*P*_peak_). Patients in *ALL2* appeared to have lower levels of inflammation and required less intensive ventilatory support. Cluster *ALL3* exhibited higher fibrinogen, CRP, and lower PCT values. Patients in *ALL3* also required more intensive ventilatory support. Visualization using the low dimensional representation uniform manifold approximation and projection (UMAP) in Supplementary Figure E11 suggested some overlap among clusters, indicating gradual transitions between subgroups rather than discrete separations. An outlier group was noted especially in the ECMO category, characterized by high ECMO pump speeds attributed to device-specific variations. To assess the robustness of the clustering method, analyses were repeated with varying random seeds. Supplementary Figure E12 and Table E3 illustrate the high robustness of the clustering results, with mean adjusted Rand index values of ≥0.98 across 50 random seeds and ≥0.93 across 100 subsampling iterations using 80% of the data. This confirms that the cluster assignments are robust and reproducible, and are unaffected by initialisation or perturbation.

### Cluster analysis on further parameter categories

**Figure 3:**
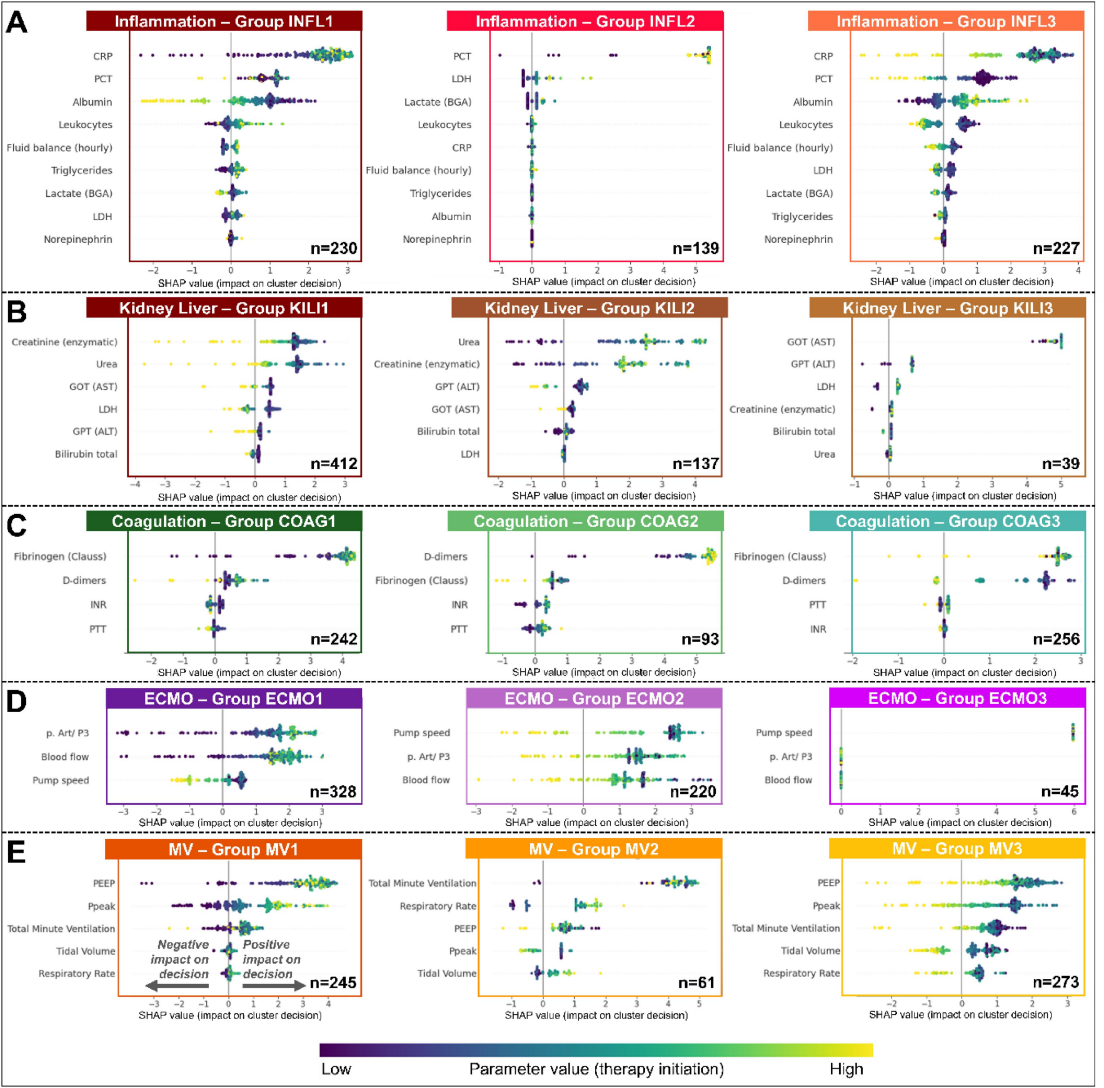
Ranking clinical parameters by their impact on the cluster assignment from low values (blue) to high values (yellow) using SHAP values for the categories inflammation (A), kidney and liver (B), coagulation (C), ECMO (D) and MV (E). Minor sample size variations across categories are due to missing parameter data in specific domains.

Clustering based on inflammation-related parameters identified three groups. As shown in Figure 3A the cluster group *INFL1* consisted of patients with higher CRP and lower PCT and albumin levels. Cluster *INFL2* was mainly characterized by high PCT. Cluster *INFL3* displayed low CRP and low PCT levels. As shown in Supplementary Figure E10 the parameters PCT and CRP were the key factors influencing inflammation cluster assignment and their relationship can be seen in Supplementary Figure E13 in panel A. In the low-dimensional representation, the *INFL3* group with a low inflammatory response is largely delimited while the other clusters show transitions that reflect the spectrum of inflammatory responses.

Clustering based on kidney and liver function parameters identified cluster group *KILI1* with lower creatinine and urea levels (Figure 3B). Cluster *KILI2* was characterized by relatively high urea and creatinine levels. Cluster *KILI3*, a smaller group distinguished by markedly elevated glutamic-oxaloacetic transaminase (GOT) levels, indicated hepatic dysfunction. A clear differentiation was observed between clusters, particularly between *KILI1* and the others.

Cluster group *COAG1* of coagulation parameters was characterized by higher fibrinogen levels and lower D-dimer concentrations (Figure 3C). Cluster *COAG2* displayed high D-dimer levels, indicating increased fibrinolytic activity. Cluster *COAG3* exhibited lower fibrinogen and D-dimer levels. Supplementary Figure E13 panel B illustrates the distinct positioning of clusters based on D-dimer and fibrinogen levels, confirming the differentiation among coagulation clusters.

Figure 3D illustrates that, among ECMO parameters, cluster group *ECMO1* consisted of patients with high pump speeds and blood flow rates, indicating higher ECMO support levels. Cluster *ECMO2* had lower pump speeds and pressures (p. Art/P3). Cluster *ECMO3* was distinguished by differences in pump speed, attributed to device-specific variations (e.g., different ECMO machines like Cardiohelp vs. DP3 systems). This cluster was clearly separated from the others due to the influence of pump speed on cluster formation.

Among MV parameters, cluster group *MV1* is characterized by patients with relatively high PEEP and P_peak_ as shown in Figure 3E. Cluster *MV2* was characterized by higher total minute volumes and respiratory rates. Cluster *MV3* showed lower PEEP, P_peak_, and overall less intensive ventilation, potentially reflecting a lung-protective strategy or patients with less severe lung injury.

### Assessment of survival factors and clinical outcomes

**Figure 4:**
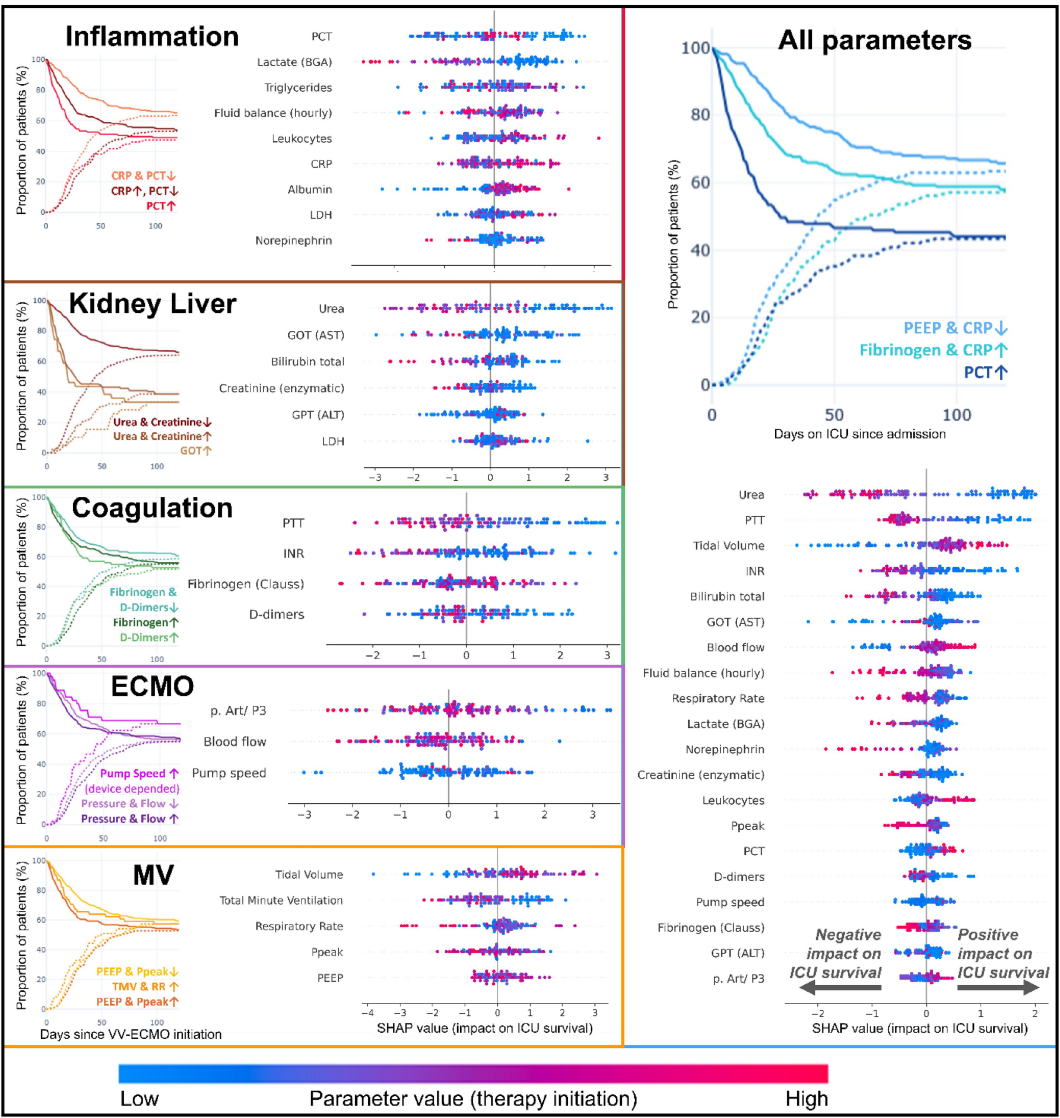
This figure highlights the key factors influencing ICU survival and the differences in survival outcomes across clinical categories. SHAP values show the impact of each parameter on survival, with positive SHAP values indicating an increased likelihood of survival and negative values showing a decreased likelihood. The colour coding, from blue (low) to red (high), represents the actual parameter values, helping to visualize how different levels influence outcomes.

**Figure 5:**
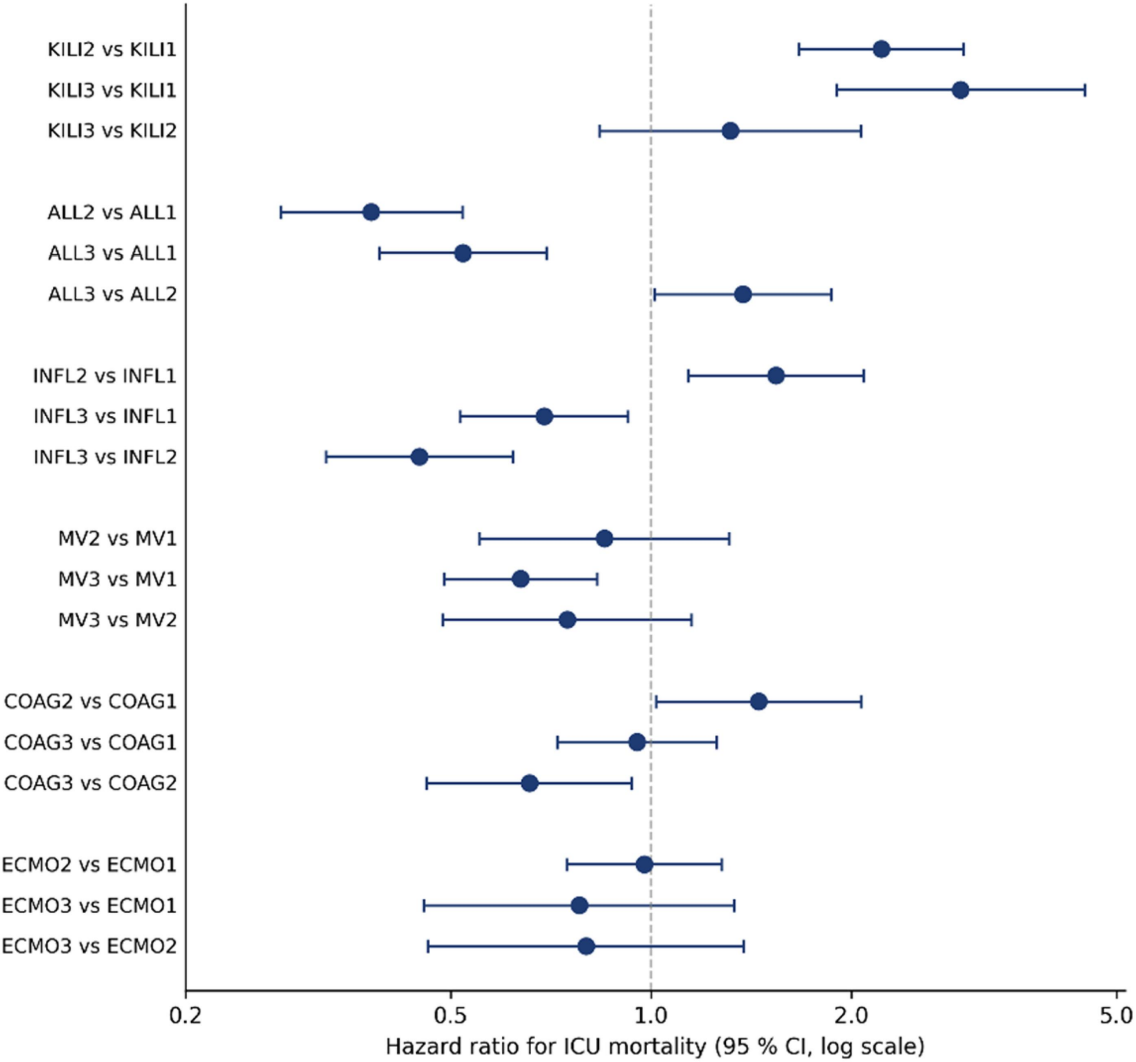
Forest plot of pairwise hazard ratios for ICU mortality across clustering categories. Each marker shows the hazard ratio (HR) from a per-category Cox proportional-hazards model adjusted for age and sex, with horizontal bars indicating the 95% confidence interval.

Figure 4 shows patient mortality and discharge for each identified cluster after ECMO initiation, and the SHAP-based contribution of clinical parameters to predicted ICU survival. The findings reveal that primary outcomes varied across the clusters, with ICU survival rates ranging from 33% to 68%. The ranking of survival differences between cluster groups was most prominent in the kidney and liver function category, where the survival difference was 32%, followed by 21% in the all-parameters category, and 17% in the inflammation category. ECMO parameters, coagulation, and mechanical ventilation settings had survival differences of 12%, 8%, and 5%, respectively. After joint Benjamini-Hochberg correction across the eighteen category-by-endpoint tests, between-cluster differences were significant at all three endpoints in the kidney/liver, all-parameters, and inflammation categories (Figure 5 and Supplementary Table E4). The strongest pairwise effect on ICU mortality was observed in the kidney/liver category (HR: 2.91, 95% CI: 1.90–4.48 for *KILI3* vs. *KILI1*). Mechanical-ventilation clusters reached significance with a smaller effect, whereas the coagulation and ECMO-settings categories showed no significant adjusted between-cluster differences.

In the all-parameters model, improved survival was linked to lower urea and partial thromboplastin time (PTT), higher tidal volume (V_T_), and reduced international normalized ratio (INR) and bilirubin. Within the inflammation category, nine parameters were identified as having a relatively balanced impact on survival, including lower levels of PCT, lactate, and triglycerides. In the kidney and liver cluster category, the most influential survival factors were elevated levels of urea, GOT, and bilirubin, indicating that renal and hepatic dysfunction had a substantial impact on survival. For the coagulation category, the influence of four parameters was relatively balanced, with lower PTT and INR being the key survival factors. In the ECMO parameters category, pressure parameters had the highest influence, with decreased arterial pressure being the main factor associated with better survival. This suggests that optimizing ECMO pressure settings could contribute to improved patient outcomes. Finally, in the mechanical ventilation settings category, V_T_ had the greatest impact on survival, with higher V_T_ values being associated with better outcomes. The influence of ventilation settings appeared to be relatively balanced.

## Discussion

In this cohort study of 598 ARDS patients treated with VV-ECMO, our primary objective was to identify and describe clinically relevant subphenotypes that could refine risk stratification and guide personalized treatment. Initially, we investigated whether clustering based on all 26 clinical parameters reflecting the initial VV-ECMO treatment phase could reveal subgroups. We identified three distinct clusters that were primarily differentiated by variations in PCT, CRP, creatinine, PEEP, and fibrinogen levels. This highlights the substantial heterogeneity within our cohort despite pneumonia being the predominant underlying condition. Notably, aside from PEEP, parameters directly related to mechanical ventilation and ECMO support contributed only moderately to cluster differentiation.

Assessing all parameters simultaneously can obscure critical clinical signals because high-dimensional data often dilutes or masks essential pathophysiological mechanisms when some parameters show limited variation or relevance. Although the methods have proven robust and reproducible, differences in the structure and quality of data among parameters can significantly influence the resulting rankings. To address this challenge, we refined our approach by preselecting sets of parameters that belong to clinically coherent domains. We focused on distinct categories such as coagulation, inflammation, kidney and liver function, ECMO settings, and mechanical ventilation. This targeted strategy allowed us to capture organ specific processes more clearly, preserve critical pathophysiological information within each domain, and improve the interpretability of our findings.

By focusing on these predefined categories, the clusters were even more differentiated. For example, in the inflammation group, PCT and CRP emerged as the most influential factors in differentiation, surpassing other markers such as lactate, and albumin. In the ECMO category, we observed a balanced distribution of parameters, including pump speed, pressures, and flow, without a single dominant parameter. However, ECMO settings were comparable in the initiation of therapy with small changes over the years in our centre, clearly avoiding extreme conditions. By contrast, mechanical ventilation clustering was mainly driven by PEEP and total minute ventilation, suggesting that the ventilatory strategy had a marked influence on subphenotype formation in our cohort.

Are these clusters clinically relevant? We examined survival rates and length of stay on ICU, which were key outcome markers that were explicitly not used in defining the clusters. Of note, outcome was not part of the clustering algorithm. However, the ICU survival outcomes observed among the clusters ranged from 33% to 67%, highlighting considerable heterogeneity in patient trajectories. This variability indicates that even among patients who meet conventional criteria for VV-ECMO, subgroups respond differently.

In the kidney and liver cluster category, we observed the widest survival gradient, with differences of up to 32% between clusters, demonstrating that dysfunction in these organ systems has critical prognostic significance. Clustering based on all parameters also revealed survival differences of up to 21% between groups, which reinforces the concept that distinct biological trajectories exist among patients who appear uniformly severe by traditional clinical criteria. In the inflammation category, a 17% difference in survival, driven by PCT and CRP, further supports previous evidence that hyperinflammatory states can worsen lung injury and multi-organ dysfunction.

In contrast, the survival differences observed in clusters based on ECMO settings, coagulation status, and mechanical ventilation were relatively modest. The association of higher tidal volumes in the mechanical ventilation category with better survival seems to contradict established low *V_T_* ventilation principles (33). However, most *V_T_* in our study fell between 160 mL and 282 mL, and high *V_T_* may still be far below 6 mL/kg. We interpret this absolute range as an indicator of preserved lung compliance and less severe injury. We note that reductions below the physiological dead space are unlikely to improve outcomes, but instead reflect disease severity. Similarly, the minor variations in ECMO parameters appear to arise from the implementation of uniform treatment protocols within our institution. The cluster *ECMO3* reflects device-related differences in pump speed between the ECMO platforms used in our centre, rather than representing a physiological subgroup. Overall, these observations are consistent with prior evidence in ARDS that certain laboratory and physiological profiles predict differential responses to therapies such as higher PEEP, prone positioning, and fluid management.

Recent research on ARDS phenotyping has increasingly highlighted the importance of systemic inflammation and organ dysfunction in determining clinical trajectories. Calfee et al. identified two ARDS phenotypes: hyperinflammatory and hypoinflammatory. The former type is characterised by high cytokine levels, shock and higher mortality (19–21). Similarly, clusters *INFL2* and *ALL1* exhibited a hyperinflammatory profile due to their high PCT and CRP levels, whereas our low-inflammation clusters reflected the hypoinflammatory group. The study by Liu et al. identified a metabolic-renal subtype that was associated with the lowest ICU survival rate (22). The significant mortality gap observed between clusters in the kidney and liver categories seems consistent with higher mortality in multi-organ failure. Phenotypes derived from non-ECMO cohorts may not be automatically applicable since extracorporeal circulation changes cytokine kinetics, coagulation and ventilator dependence. These therapy-induced changes can impact the prognostic signals important after cannulation, requiring subphenotyping to be recalibrated during ECMO.

Nishikimi et al. extended phenotyping to an ECMO setting, incorporating chest computed tomography (CT) to define dry, wet, and fibrotic types. They showed that only the oedematous wet group benefited from higher PEEP (25). However, CT scans in ECMO patients cannot be applied in every setting. Therefore, we focused on data applicable in nearly all settings, namely routinely recorded numerical data. However, there is emerging evidence that CT patterns, such as extensive consolidation or the Macklin sign, can predict barotrauma and failed liberation (34, 35). Future research may further focus on integrating structured imaging into multimodal approaches.

Together, these studies and our results suggest that inflammatory, renal, and hepatic markers continue to be reliable risk indicators after cannulation, which may also be driven by complications of the extracorporeal circuit. Formal between-cluster outcome inference (Figure 5 and Supplementary Table E4) confirms this pattern, with adjusted differences in ICU mortality, ICU length of stay, and ECMO duration concentrated in the kidney/liver, all-parameters, and inflammation categories. Mechanical-ventilation clusters reached significance with a smaller effect, whereas coagulation and ECMO-settings categories showed no adjusted between-cluster differences, consistent with the limited spread of these parameters in our centre. Overall, by describing the interplay during ECMO between organ dysfunction, inflammation and mechanical support strategies during ECMO therapy, our targeted analysis approach adds granularity to the phenotypic classification of ARDS.

Furthermore, these cluster groups and time-stamped EHRs provide a structured approach to causal and early treatment response analyses. With these foundations, future studies may generate hypotheses and emulate differential treatment effect trials, with the overarching aim of translating the clusters into phenotype-driven therapy. In practice, data-driven profiles could inform bedside decision support, for example by prioritising patients with hyperinflammation (*INFL2*) or kidney/liver dysfunction (*KILI2*, *KILI3*) for enhanced organ support. Target trial emulation and other causal frameworks can evaluate the impact of early interventions on phenotype transitions and outcomes (36, 37). These cluster groups and datasets, derived from time-stamped EHRs and including critical laboratory markers and machine parameters, could support the development of phenotype-driven therapeutic devices.

Several limitations must be acknowledged. Firstly, this study was conducted in a single high-volume ARDS and ECMO centre, which may restrict its generalisability. The centre implements standardised VV-ECMO initiation criteria and targets specific physiological ranges, so parameters such as ECMO and ventilation settings may display less variability than in broader multicentre cohorts. The dataset excludes patients who met VV-ECMO eligibility but were ultimately managed without cannulation at referring hospitals, introducing selection bias and limiting applicability to the full ARDS population. Since all ECMO and ventilation parameters were captured after cannulation these parameters cannot guide pre-ECMO selection and future work must incorporate pre-cannulation data to test generalisability.

Additionally, retrospective data processing introduces methodological constraints. Although our workflow ensures it captures the initial stabilisation phase, precise temporal anchoring of the 48-hour observation window was limited by documentation variability. Furthermore, to avoid model-based correlations and ensure equal parameter contributions, we employed median imputation and min-max scaling. In K-means, the spherical and equal-variance assumptions represent a methodological limitation that was pragmatically addressed through range equalisation. Although Winsorisation prior to this and low missingness had mitigated the risk of distortion, they could still cause variance shrinkage or compress the distributions of skewed variables. Future studies could explore cluster robustness using alternative processing strategies such as multiple imputation, robust scaling, and clustering algorithms. The selection of parameters was limited by the availability and quality of the data. For example, some extended haemodynamic markers could not be included, which prevented an assessment of right ventricular dysfunction. Although robust clustering algorithms were used, there remains a potential for classification bias due to the inherent complexity, quality and missingness of EHR data (38). Furthermore, heterogeneity across ECMO platforms could not be separately quantified within the routine-EHR scope of the study. Additionally, SHAP attributions for cluster assignments are model-dependent associations, and their interpretability and consistency may be further constrained by the complexity of the model. Finally, factors such as referral bias, comorbidities, and centre-specific practices could influence cluster composition. The proportional-hazards assumption was not satisfied in two cluster categories (all-parameters and kidney/liver). Their hazard ratios are therefore interpreted as average effects over follow-up, complemented by the cluster-stratified time course shown in Figure 4. Estimates are least precise for the smallest individual clusters, *KILI3* within the kidney/liver category (*n* = 39) and *ECMO3* within the ECMO-settings category (*n* = 45).

Formal validation requires a stepwise approach. Firstly, these findings must be confirmed and heterogeneity across healthcare settings accounted for by external validation in public registries (e.g., ELSO) and multicentre studies that enrol every eligible cannulated patient and include comprehensive pre- and post-cannulation parameters. Secondly, prospective cohorts that assign subphenotypes post-cannulation and monitor predefined outcomes are needed. Thirdly, phenotype-stratified interventional studies could determine the benefits of differential treatment.

However, this study demonstrates that cluster analysis can reveal clinically meaningful state descriptions and subphenotypes in ARDS patients treated with VV-ECMO. Our findings suggest that renal and hepatic dysfunction, coagulation abnormalities, and elevated inflammatory markers at therapy initiation are particularly prognostic. In summary, our study provides concrete evidence that targeting organ-specific dysfunctions can enhance post-cannulation risk stratification and guide the tailoring of management strategies for critically ill ARDS patients on VV-ECMO.

## Data Availability

The datasets presented in this article are not readily available because the datasets used and analysed during the current study are confidential electronic health records and cannot be shared publicly due to patient privacy regulations. Requests to access the datasets should be directed to Christian Karagianndis, karagiannidisc@kliniken-koeln.de.
