# Supplementary material for: Data-driven subphenotyping of severe ARDS patients requiring VV-ECMO
Source: Front Digit Health. 2026 Jul 3;8:1736849. doi: 10.3389/fdgth.2026.1736849 (PMC13375777; doi:10.3389/fdgth.2026.1736849)
Supplement: Supplementary file 1 [file Datasheet1.docx]

**Data-driven subphenotyping of severe ARDS patients requiring VV-ECMO**

Micha Landoll^1,2,3^, Stephan Strassmann^1^, Wolfram Windisch^1,4^, Ulrich Steinseifer^2^, Andreas Schuppert^3,5^, Michael Neidlin^2,5†^, Christian Karagiannidis^1,4†^

**Online Data Supplement.**

# Supplementary Information

**
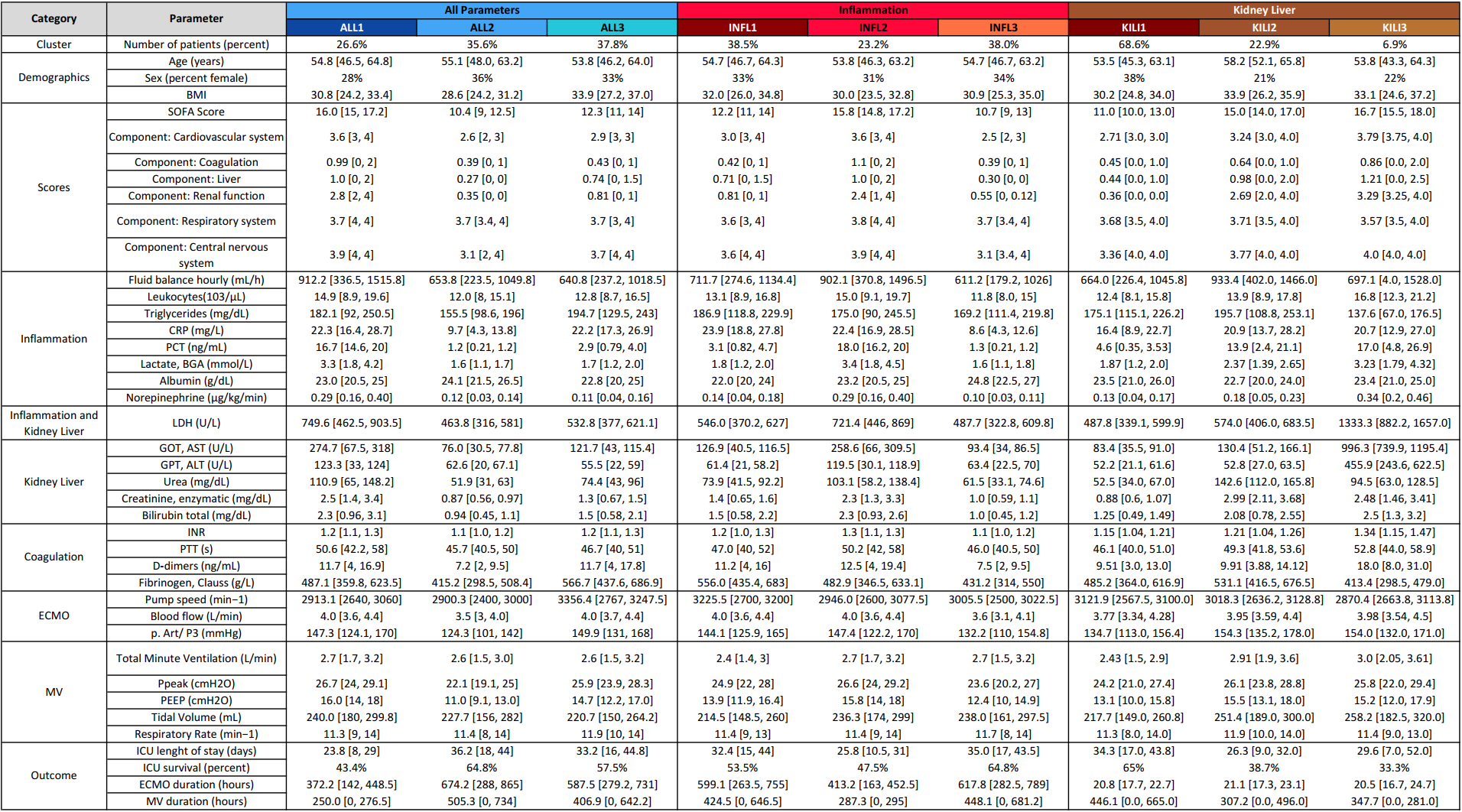
Table E1:** Parameters captured during the first 48 hours of VV-ECMO of the cluster of categories All Parameters, Inflammation, Kidney and Liver. Continuous variables are displayed as the mean, 25th and 75th percentiles, while Boolean variables are presented as percentages.


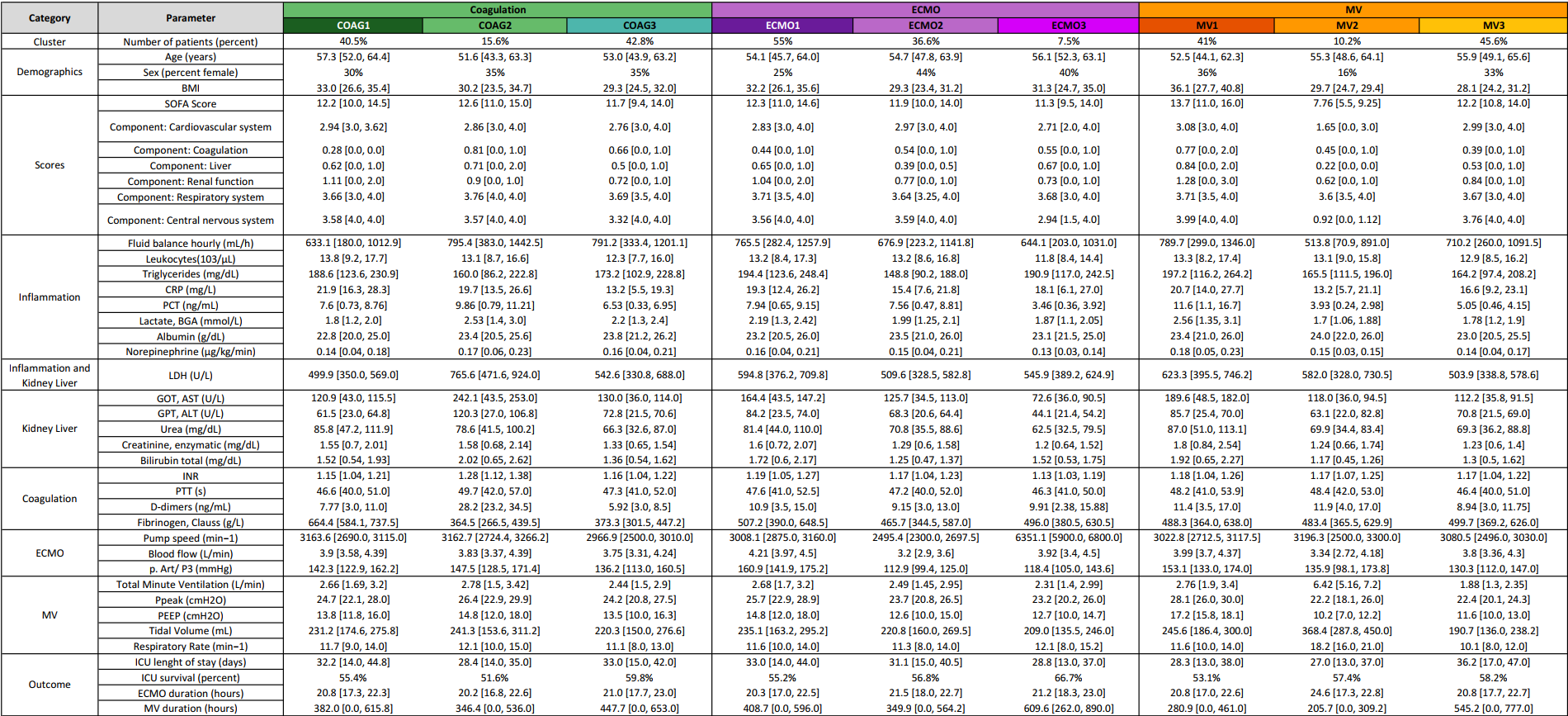
**Table E2:** Parameters captured during the first 48 hours of VV-ECMO of the cluster of categories Coagulation, ECMO and MV. Continuous variables are displayed as the mean, 25th and 75th percentiles, while Boolean variables are presented as percentages

| **Cluster category** | **Mean ARI for K-means robustness with seed variation** | **Mean ARI for K-means robustness with resampling** |
| --- | --- | --- |
| All Parameters | 0.9998 | 0.9318 |
| Inflammation | 1 | 0.9616 |
| Kidney Liver | 1 | 0.9889 |
| Coagulation | 0.9855 | 0.9504 |
| ECMO | 1 | 0.9887 |
| MV | 0.9991 | 0.931 |

**Table E3**: This table summarizes robustness analyses for K-means across the cluster categories. Seed stability was assessed with adjusted Rand indices (ARI) over 50 random initialisations (mean ARI ≥ 0.99), while resampling robustness used 100 iterations with 80% resampling.

| Cluster | Number of patients | ICU survival, % | Hazard Ratio (95 % CI) | p value (Wald) | Median ICU LOS, days (95 % CI) | Median ECMO duration, days (95 % CI) |
| --- | --- | --- | --- | --- | --- | --- |
| Kidney/liver. Cox LR p_BH = 1.2e-8, KW ICU LOS p_BH = 6.5e-5, KW ECMO duration p_BH = 1.8e-6, alive-discharge cause-specific log-rank p = 0.13, Schoenfeld Bonferroni p = 8.7e-4 [PH violated]. | | | | | | |
| *KILI1* | 412 | 65.0 | 1.00 (reference) | — | 26.5 (24.5–29.0) | 18.6 (17.0–20.8) |
| *KILI2* | 137 | 38.7 | 2.21 (1.67–2.94) | 3.7e-8 | 19.0 (16.0–23.0) | 13.9 (11.6–15.9) |
| *KILI3* | 39 | 33.3 | 2.91 (1.90–4.48) | 1.1e-6 | 18.0 (10.0–26.0) | 13.0 (9.9–18.0) |
| All parameters. Cox LR p_BH = 5.2e-8, KW ICU LOS p_BH = 8.1e-8, KW ECMO duration p_BH = 3.9e-10, alive-discharge cause-specific log-rank p = 0.51, Schoenfeld Bonferroni p = 2.5e-5 [PH violated]. | | | | | | |
| *ALL1* | 159 | 43.4 | 1.00 (reference) | — | 18.0 (15.0–20.0) | 11.3 (9.0–13.5) |
| *ALL2* | 213 | 64.8 | 0.38 (0.28–0.52) | 1.5e-9 | 28.0 (24.0–32.0) | 19.9 (17.2–22.8) |
| *ALL3* | 226 | 57.5 | 0.52 (0.39–0.70) | 1.1e-5 | 25.5 (23.0–28.5) | 17.6 (16.6–19.7) |
| Inflammation. Cox LR p_BH = 1.9e-5, KW ICU LOS p_BH = 1.6e-5, KW ECMO duration p_BH = 2.7e-7, alive-discharge cause-specific log-rank p = 0.33, Schoenfeld Bonferroni p = 0.13. | | | | | | |
| *INFL1* | 230 | 53.5 | 1.00 (reference) | — | 25.0 (23.0–28.0) | 17.9 (16.7–20.1) |
| *INFL2* | 139 | 47.5 | 1.54 (1.14–2.08) | 5.2e-3 | 18.0 (15.0–20.0) | 11.8 (9.9–13.9) |
| *INFL3* | 227 | 64.8 | 0.69 (0.52–0.92) | 0.01 | 28.0 (24.0–31.0) | 17.9 (16.0–21.0) |
| Mechanical ventilation. Cox LR p_BH = 5.2e-3, KW ICU LOS p_BH = 1.5e-3, KW ECMO duration p_BH = 7.1e-6, alive-discharge cause-specific log-rank p = 0.06, Schoenfeld Bonferroni p = 0.06. | | | | | | |
| *MV1* | 245 | 53.1 | 1.00 (reference) | — | 23.0 (20.0–25.0) | 14.9 (13.8–16.8) |
| *MV2* | 61 | 57.4 | 0.85 (0.55–1.31) | 0.46 | 19.0 (16.0–26.0) | 14.0 (11.1–16.8) |
| *MV3* | 273 | 58.2 | 0.64 (0.49–0.83) | 8.2e-4 | 28.0 (24.0–31.0) | 20.4 (17.5–22.8) |
| Coagulation. Cox LR p_BH = 0.10, KW ICU LOS p_BH = 0.39, KW ECMO duration p_BH = 0.53, alive-discharge cause-specific log-rank p = 0.67, Schoenfeld Bonferroni p = 0.50. | | | | | | |
| *COAG1* | 242 | 55.4 | 1.00 (reference) | — | 26.0 (23.0–29.0) | 17.0 (16.0–19.7) |
| *COAG2* | 93 | 51.6 | 1.45 (1.02–2.07) | 0.04 | 22.0 (18.0–25.0) | 15.0 (12.9–16.9) |
| *COAG3* | 256 | 59.8 | 0.95 (0.72–1.25) | 0.73 | 24.0 (22.0–26.5) | 16.9 (15.7–18.7) |
| ECMO settings. Cox LR p_BH = 0.71, KW ICU LOS p_BH = 0.87, KW ECMO duration p_BH = 0.71, alive-discharge cause-specific log-rank p = 0.18, Schoenfeld Bonferroni p = 0.09. | | | | | | |
| *ECMO1* | 328 | 55.2 | 1.00 (reference) | — | 24.0 (22.0–27.0) | 16.6 (15.0–17.6) |
| *ECMO2* | 220 | 56.8 | 0.98 (0.75–1.27) | 0.86 | 25.0 (22.0–27.0) | 17.1 (15.1–19.9) |
| *ECMO3* | 45 | 66.7 | 0.78 (0.46–1.33) | 0.36 | 23.0 (17.0–30.0) | 16.6 (13.8–29.0) |

**Table E4**: Formal between-cluster ICU outcome inference per cluster category. Hazard ratios (HR) for ICU mortality were derived from a Cox proportional-hazards model fitted separately per cluster category and adjusted for age and sex, with the numerically first cluster (KILI1, ALL1, INFL1, MV1, COAG1, ECMO1) serving as the within-category reference. The Cox likelihood-ratio test (LR) for ICU survival and the Kruskal-Wallis (KW) tests for ICU length of stay (LOS) and ECMO duration form a multiplicity family of eighteen tests (six cluster categories times three endpoints), jointly corrected with the Benjamini-Hochberg (BH) procedure. Median ICU length of stay and median ECMO duration are reported with bootstrap 95% confidence intervals of the median. The cause-specific log-rank p-value is given for alive ICU discharge only because every patient reaches either ICU death or alive ICU discharge during follow-up, so the death-cause log-rank is identical to the standard log-rank. The Bonferroni-aggregated global Schoenfeld residual p-value tests the Cox proportional-hazards assumption. Cluster categories in which the assumption is rejected are marked [PH violated].

*
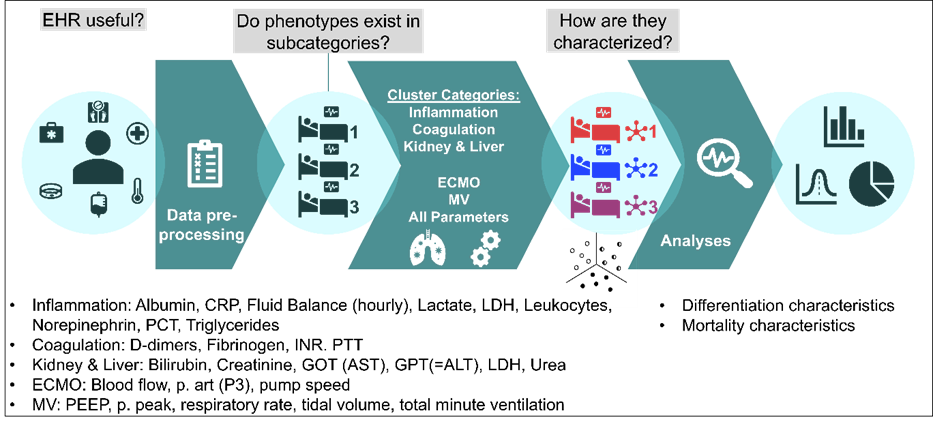
*

**Figure E1:** This figure illustrates the structured workflow of the study, aimed at identifying clinically meaningful subphenotypes of ARDS patients undergoing VV-ECMO. The process begins with systematic preprocessing of electronic health records to ensure data reliability and representativeness. The next step involves applying clustering techniques to identify distinct subphenotypes across several clinical categories, including Inflammation, Coagulation, Kidney & Liver, ECMO, Mechanical Ventilation (MV), and All Parameters combined. Following clustering, analyses are performed to characterise each subphenotype and evaluate their respective survival outcomes.


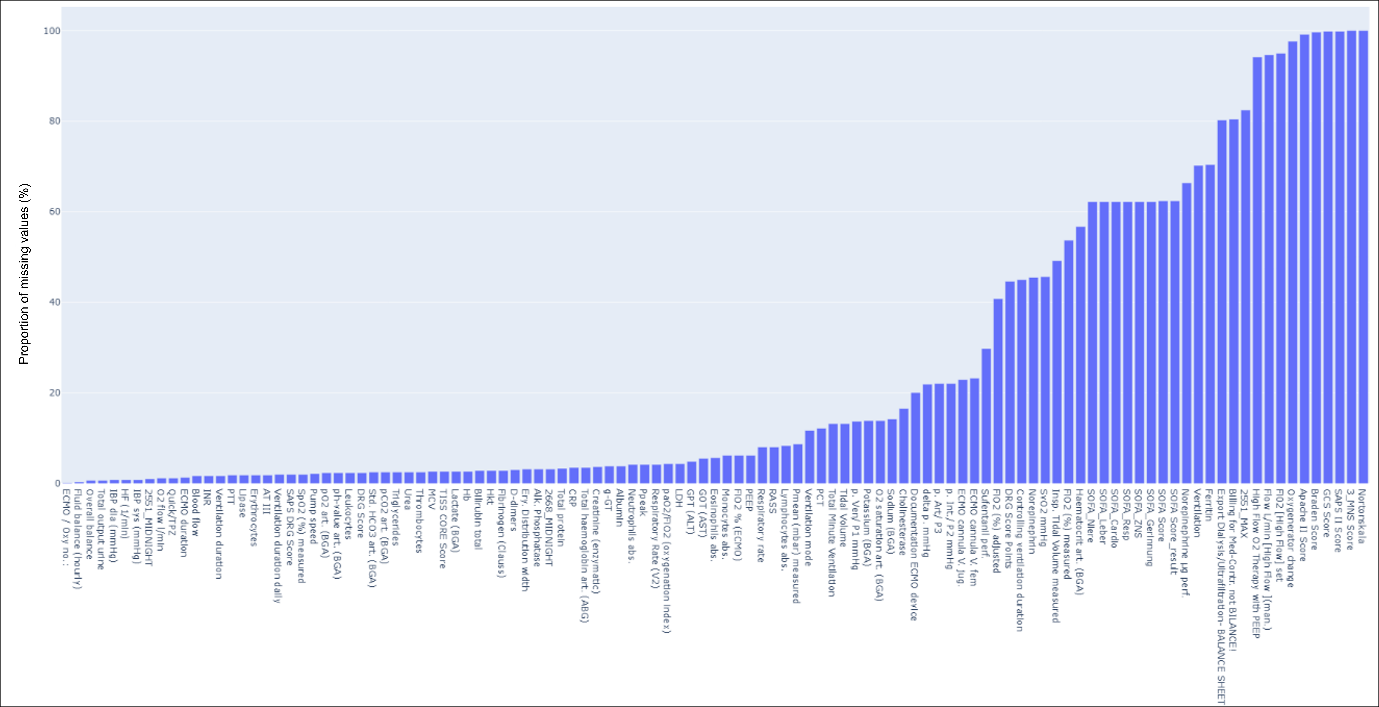


**Figure E2**: This figure displays the proportion of missing values for all available parameter in the electronic health records. Parameters with less than 23% missing data were retained for analysis, with the exception of norepinephrine with 45.5%, where missing values were imputed using median values to maintain a balance between data completeness and cohort size.


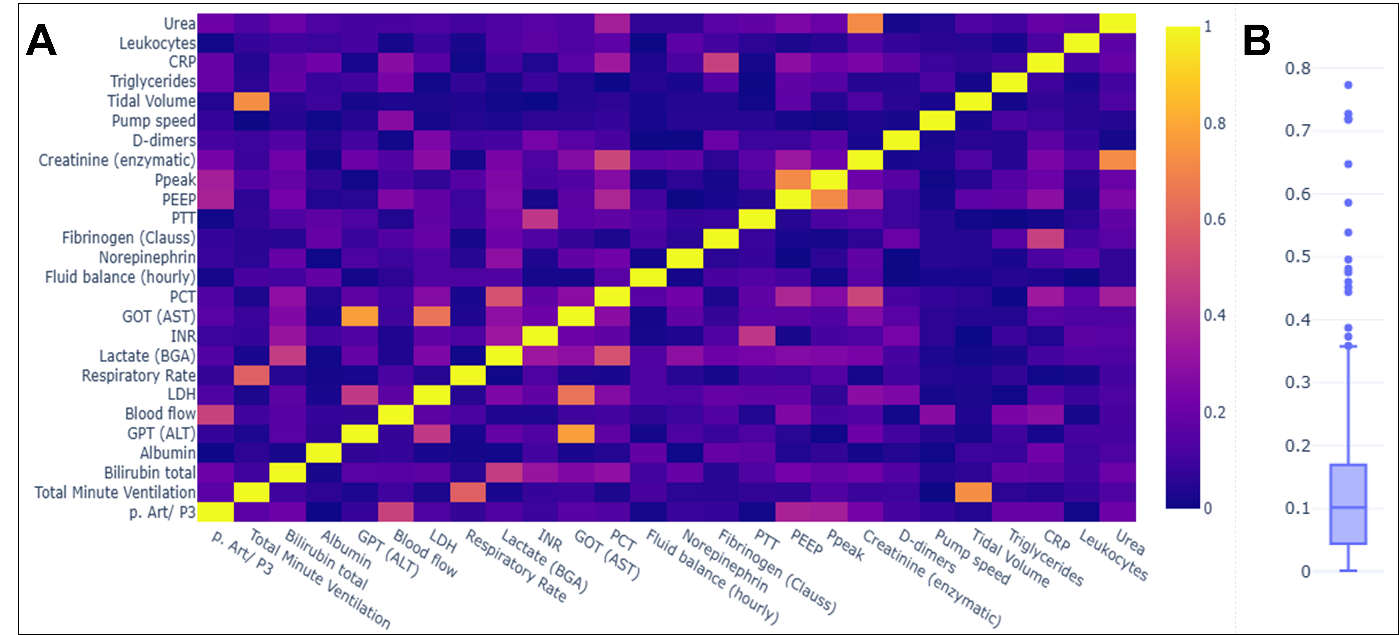


**Figure E3**: This figure shows the absolute Pearson correlation coefficients among all clinical variables as a heatmap in A, with colour intensity reflecting the strength of each pairwise relationship, and as a boxplot in B summarizing the distribution of those absolute correlations. These visualizations were used to identify and remove highly redundant parameters and retain only the most informative variables for clustering.


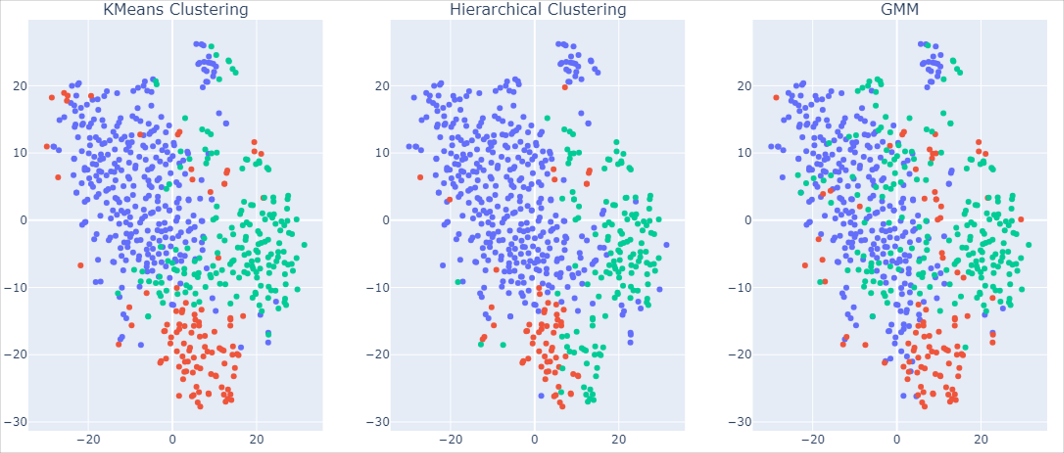


**Figure E4:** This figure compares three clustering methods applied to the all-parameters category: K-means clustering, hierarchical clustering, and Gaussian mixture models (GMM). Latent profile analysis can be conceptualized as a finite Gaussian mixture model for continuous indicators. Each method was applied to the same preprocessed dataset and visualized using t-SNE projections in low dimensions. While clustering algorithms differ in their assumptions and approaches, the visualizations demonstrate exemplary broadly consistent patient groupings, supporting the robustness of the identified subphenotypes despite methodological differences.


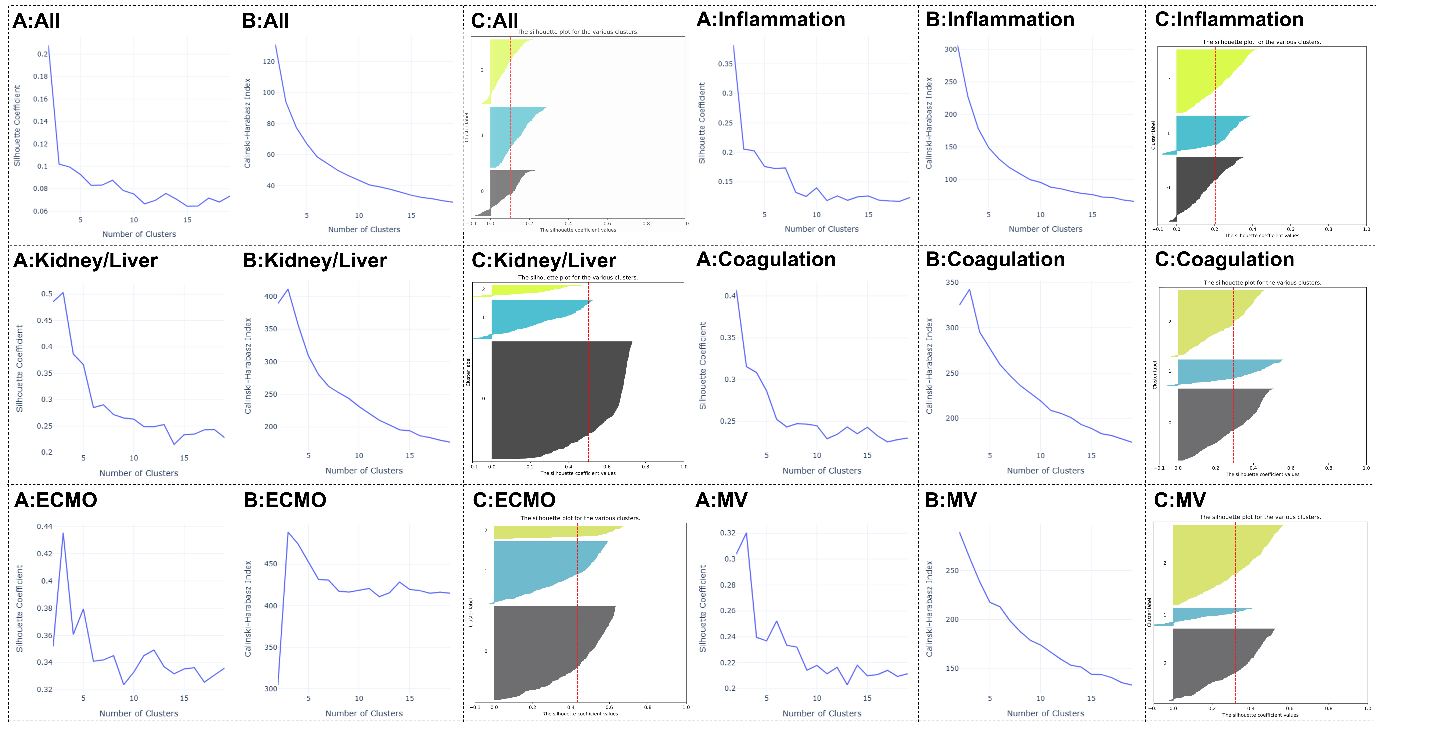


**Figure E5:** This figure presents the metrics used to determine the optimal number of clusters for different clinical categories. Panel A shows the averaged silhouette coefficients across varying numbers of clusters, Panel B displays the Calinski-Harabasz index for evaluating the cluster separation, and Panel C illustrates the silhouette coefficients for the selected number of clusters.


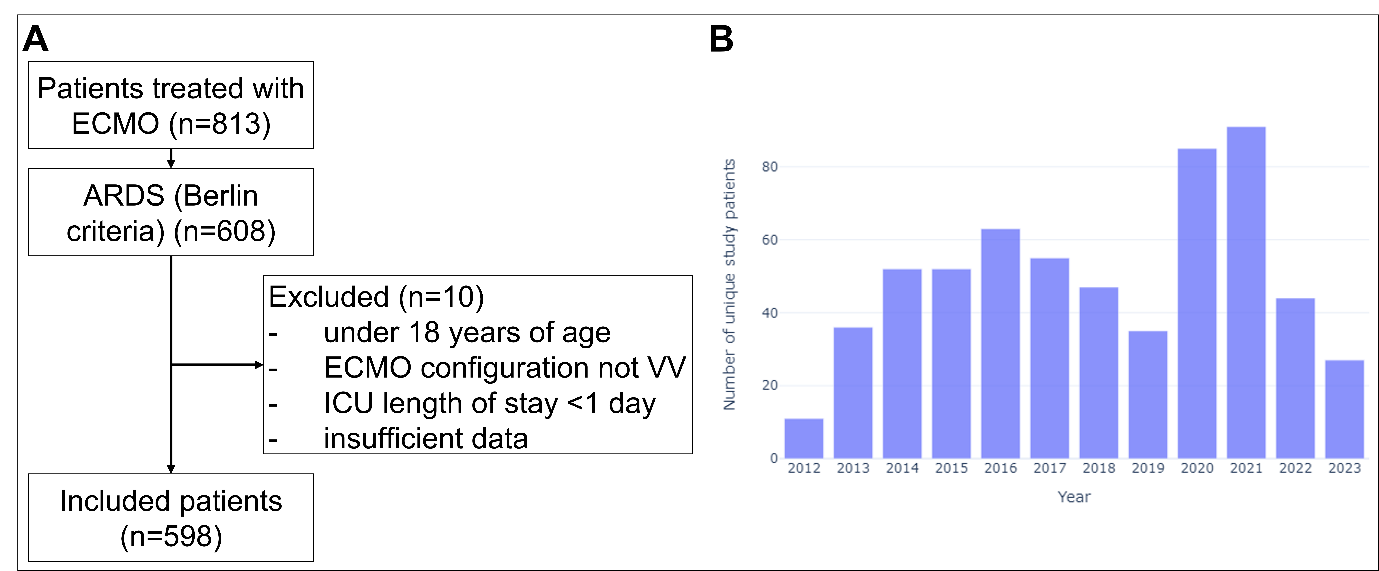


**Figure E6**: This figure provides an overview of the study cohort, including the inclusion and exclusion criteria. Panel A outlines the patient selection process, starting with initial inclusion based on ECMO treatment on ARDS diagnosis, followed by exclusions due to ICU stays shorter than one day, insufficient data, ECMO configurations other than VV, and age under 18 years. Panel B shows a bar chart illustrating the number of unique study patients per year.


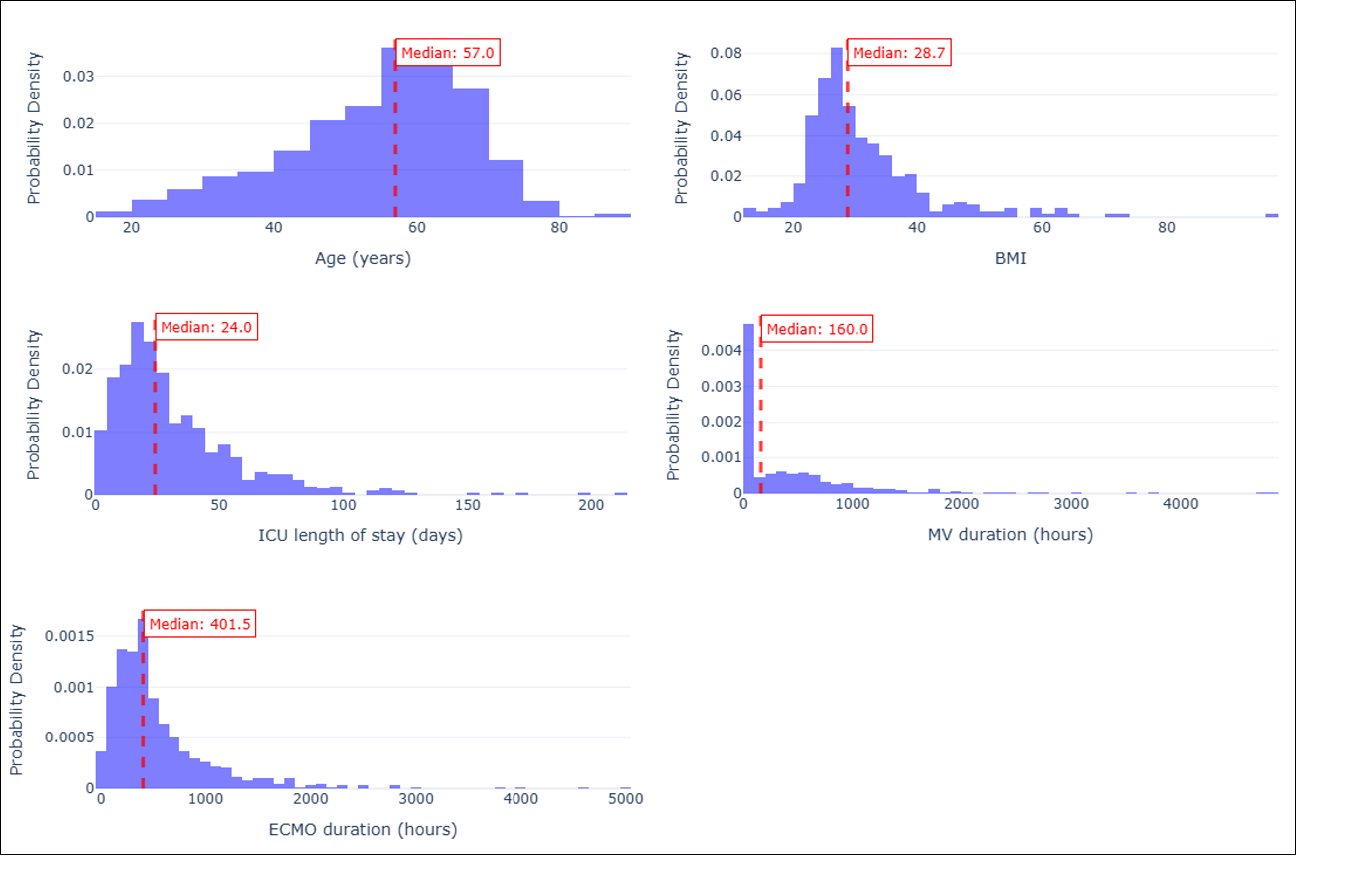
**Figure E7**: Histograms presented for age, BMI, ICU length of stay, MV duration, and ECMO duration. Red dashed lines indicate the median values.


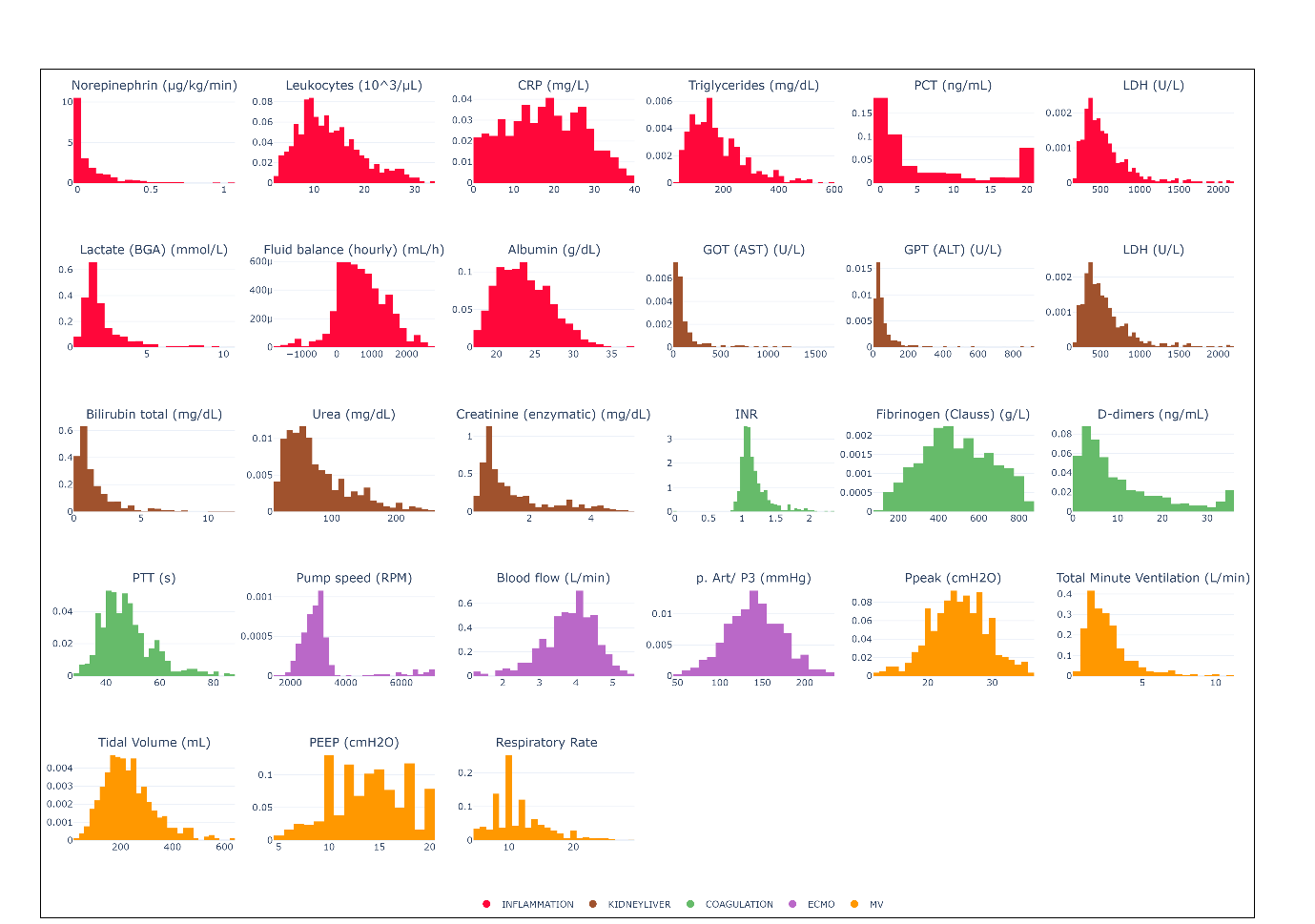


**Figure E8**: The histograms illustrate the distribution of each parameter within the study cohort, categorized into clinical groups: Inflammation (red), Kidney & Liver (brown), Coagulation (green), ECMO (purple), and MV (orange).

^^
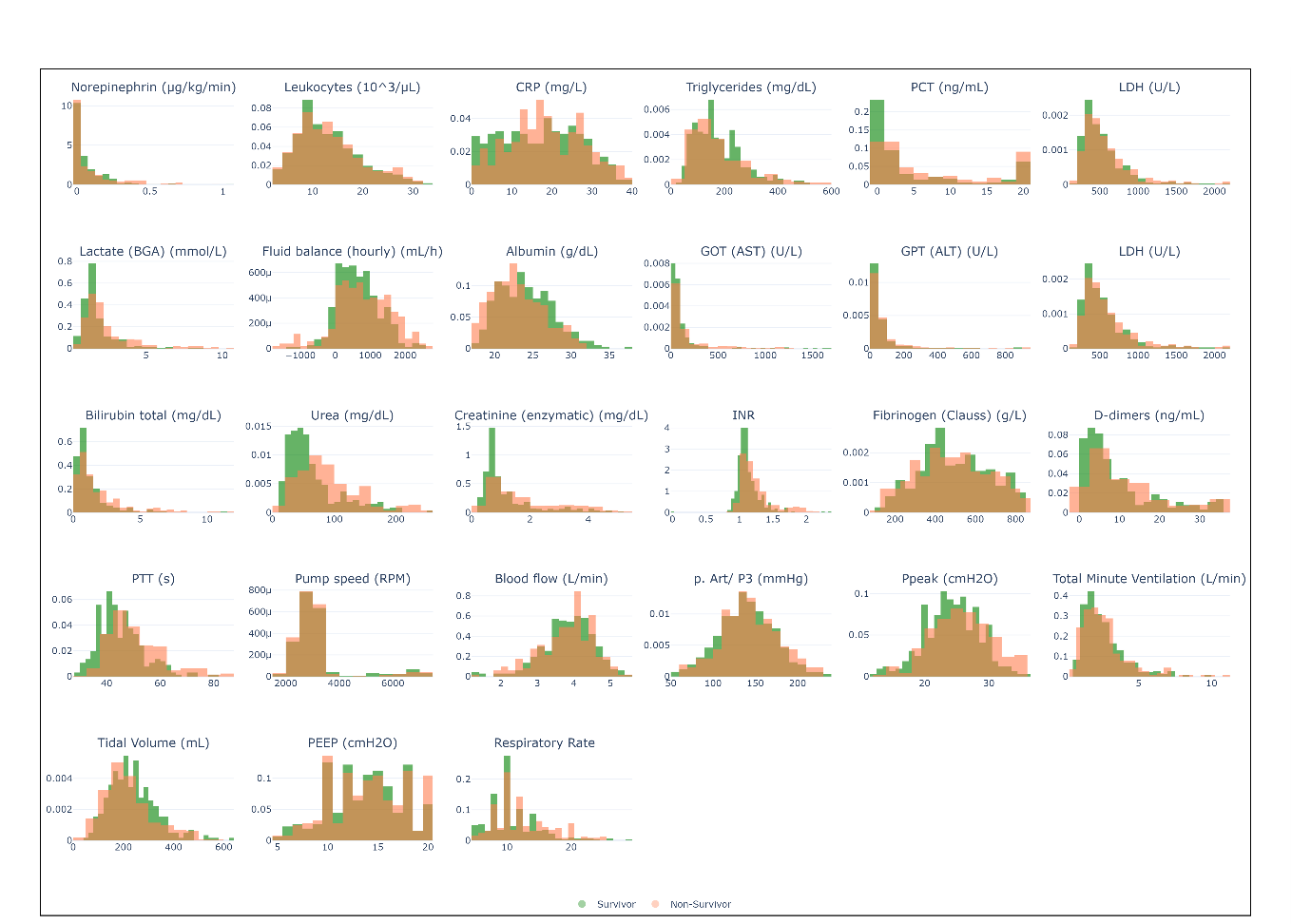


**Figure E9:** Histograms presented for the selected clinical parameters, grouped by patient survival status (survivors in green, non-survivors in red). It shows the distribution of various physiological, laboratory, and treatment-related values across both groups.


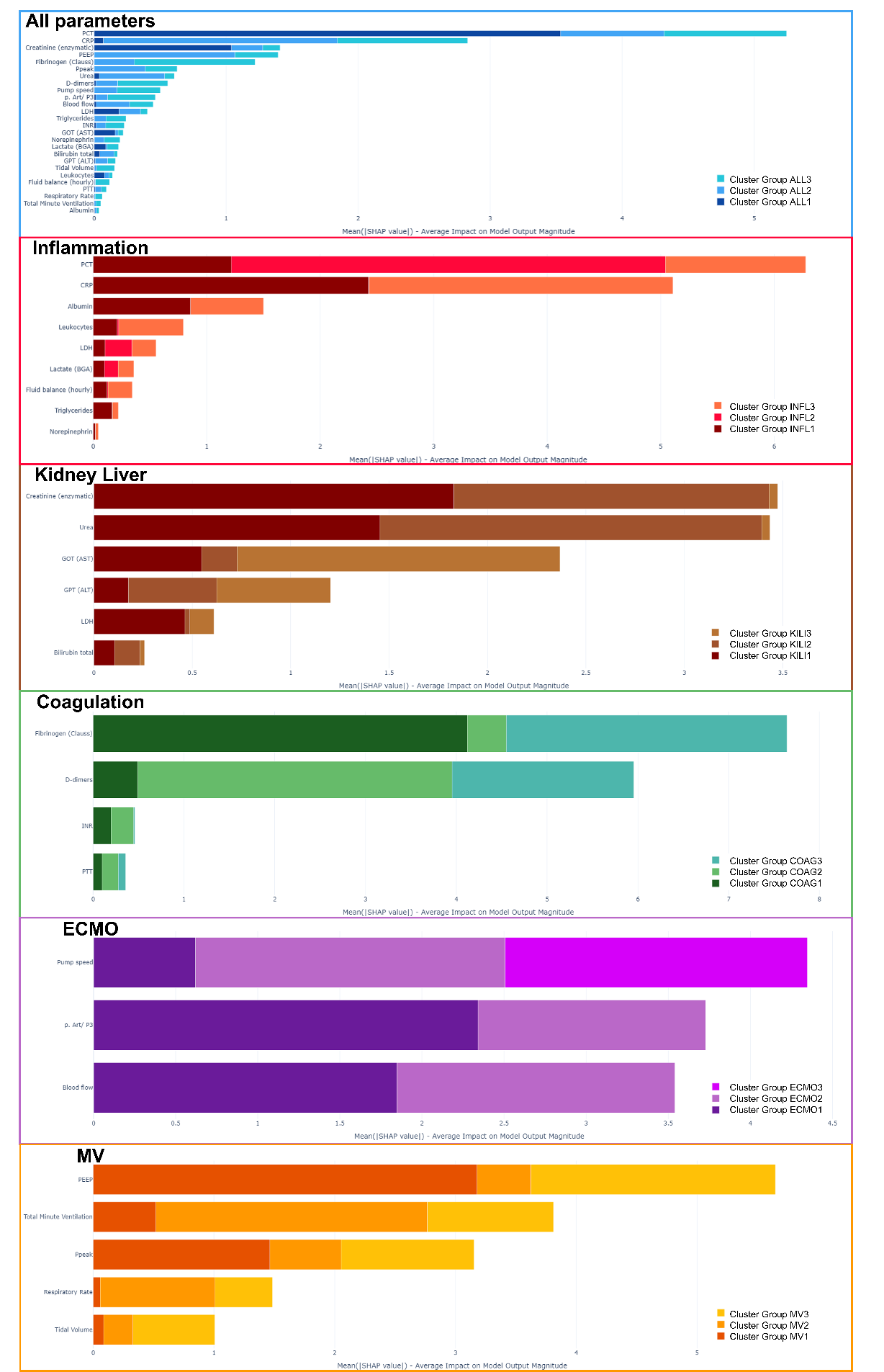


**Figure E10:** This figure summarises the SHAP values for each clinical category showing the overall importance of all cluster assignments. The length of the bars indicates the average absolute impact of each parameter on the magnitude of the model output, revealing which parameters most strongly influenced the grouping of patients within each category. For example, D-dimers and creatinine are key factors in the category including all parameters, while CRP and PCT dominate in the Inflammation category.

**
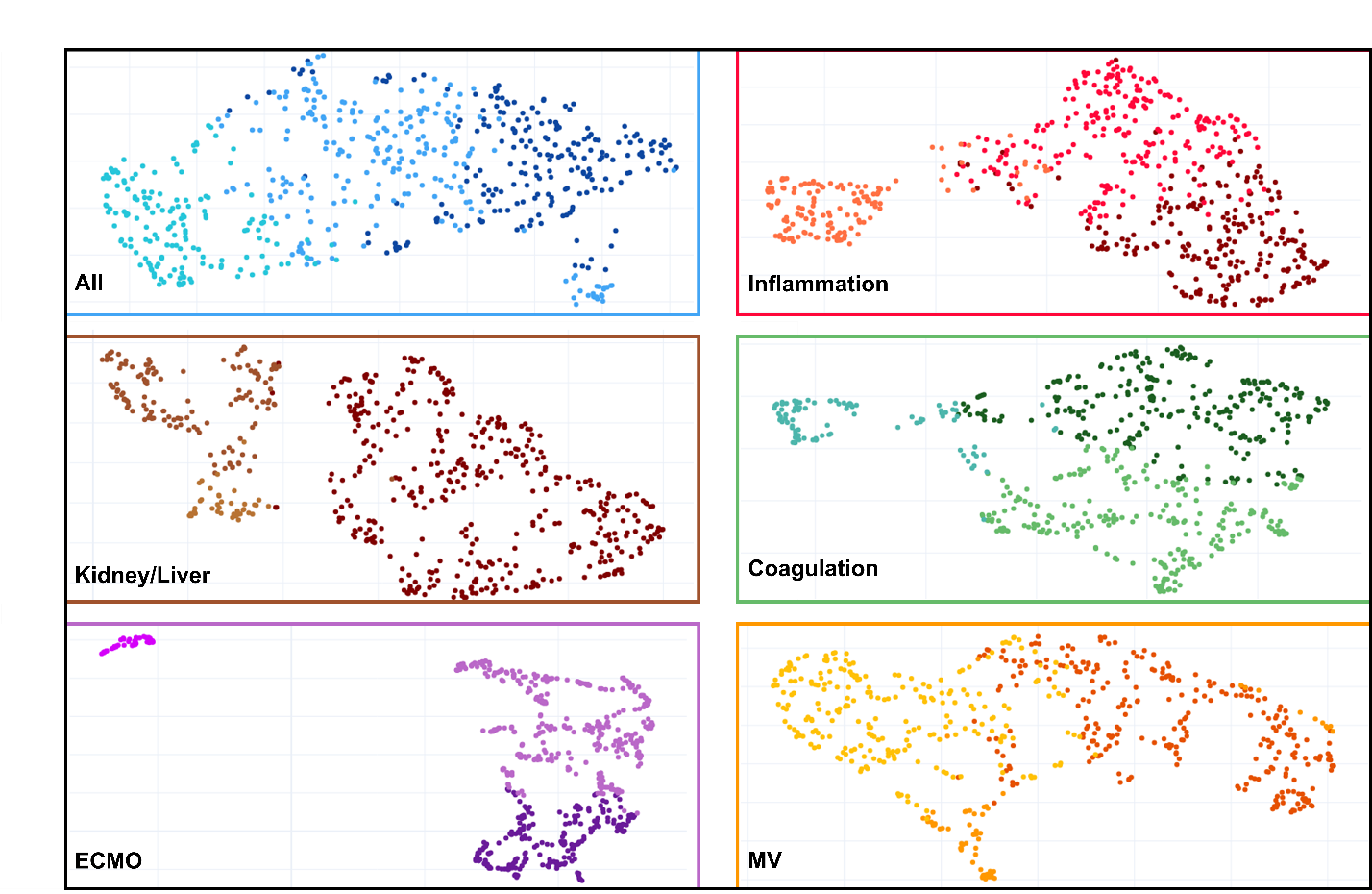
Figure E11**: This figure displays the Uniform Manifold Approximation and Projection (UMAP) representations for clusters across the clinical categories. UMAP was used to project high-dimensional data into a two-dimensional space, visualising distinct patient clusters. While some categories, such as Kidney/Liver and ECMO, show clear separation between clusters, others, like Inflammation and All Parameters, exhibit more overlap. This suggests that boundaries between some subphenotypes may be less defined and potentially transient.


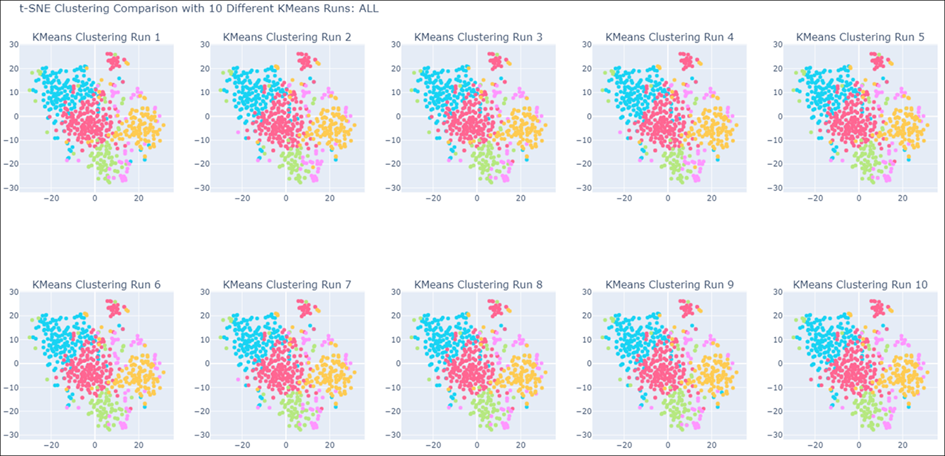


**Figure E12:** This figure illustrates the robustness of K-means clustering by comparing t-SNE projections of 10 different clustering runs on all parameters, each initiated with a different random seed. Despite variations in the random initialization, the cluster groupings remain consistent across runs, demonstrating the stability and reproducibility of the clustering method.

**
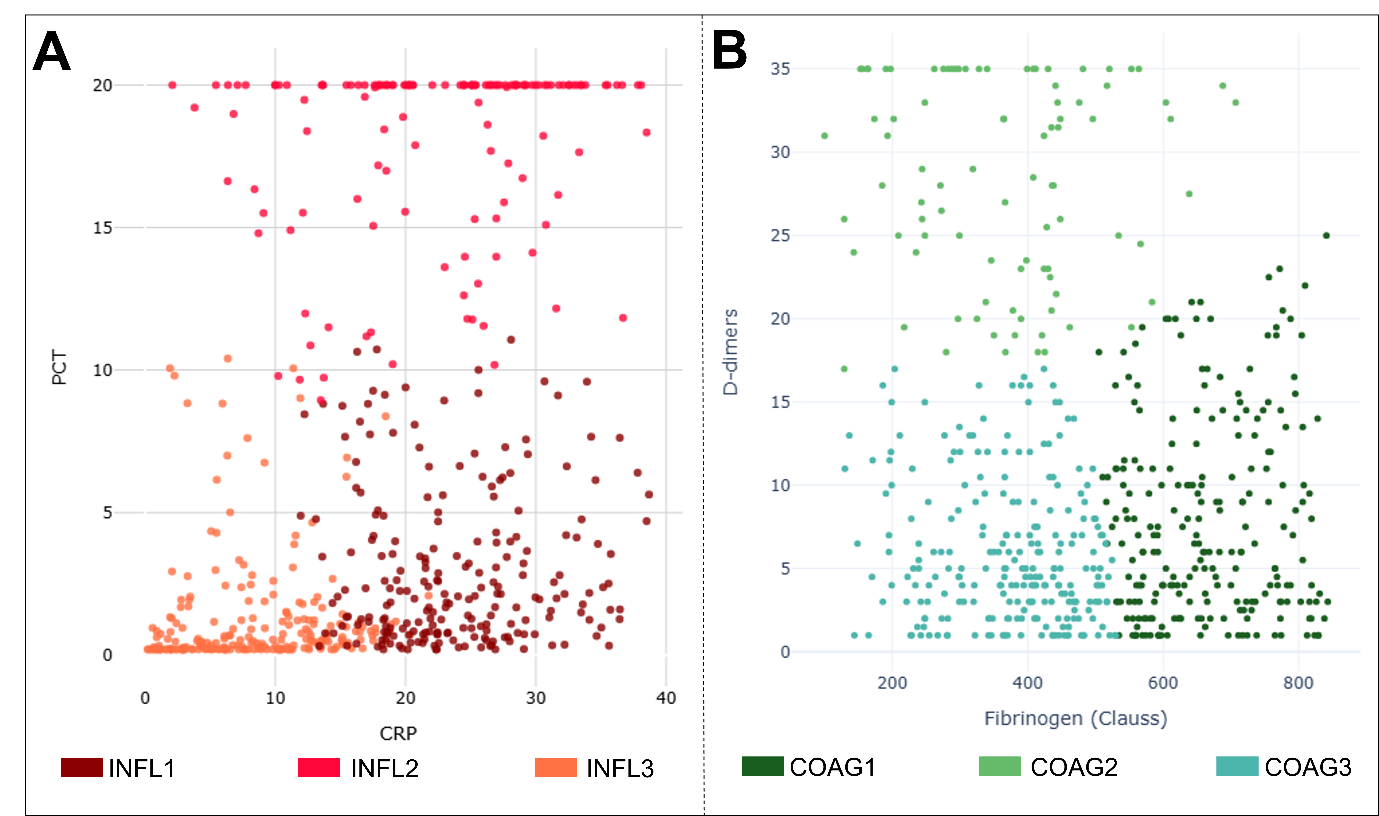
Figure E13:** Panel A plots procalcitonin versus C-reactive protein for INFL3 (orange, minimal elevations), INFL1 (dark red, elevated CRP with intermediate PCT), and INFL2 (red, saturating PCT across the CRP range). Panel B plots D-dimer versus fibrinogen for COAG3 (turquoise, low both), COAG2 (light green, intermediate) and COAG1 (dark green, high fibrinogen with broader D-dimer), revealing distinct coagulation clusters.
